# Enhancing Fatty Acids Oxidation *via* L-Carnitine Attenuates Obesity-Related Atrial Fibrillation and Structural Remodeling by Activating AMPK Signaling and Alleviating Cardiac Lipotoxicity

**DOI:** 10.3389/fphar.2021.771940

**Published:** 2021-11-26

**Authors:** Yudi Zhang, Yuping Fu, Tiannan Jiang, Binghua Liu, Hongke Sun, Ying Zhang, Boyuan Fan, Xiaoli Li, Xinghua Qin, Qiangsun Zheng

**Affiliations:** ^1^ The Second Affiliate Hospital of Xi’an Jiaotong University, Xi’an, China; ^2^ Department of Cardiology, Beijing Anzhen Hospital, Capital Medical University, Beijing, China; ^3^ School of Life Sciences, Northwestern Polytechnical University, Xi’an, China

**Keywords:** atrial fibrillation, obesity, fatty acids oxidation, lipotoxicity, l-carnitine, AMPK (5′-AMP activated kinase)

## Abstract

Atrial fibrillation (AF) is the most common sustained cardiac arrhythmia in clinical setting. Its pathogenesis was associated with metabolic disorder, especially defective fatty acids oxidation (FAO). However, whether promoting FAO could prevent AF occurrence and development remains elusive. In this study, we established a mouse model of obesity-related AF through high-fat diet (HFD) feeding, and used l-carnitine (LCA, 150 mg/kg⋅BW/d), an endogenous cofactor of carnitine palmitoyl-transferase-1B (CPT1B; the rate-limiting enzyme of FAO) to investigate whether FAO promotion can attenuate the AF susceptibility in obesity. All mice underwent electrophysiological assessment for atrial vulnerability, and echocardiography, histology and molecular evaluation for AF substrates and underlying mechanisms, which were further validated by pharmacological experiments *in vitro*. HFD-induced obese mice increased AF vulnerability and exhibited apparent atrial structural remodeling, including left atrial dilation, cardiomyocyte hypertrophy, connexin-43 remodeling and fibrosis. Pathologically, HFD apparently leads to defective cardiac FAO and subsequent lipotoxicity, thereby evoking a set of pathological reactions including oxidative stress, DNA damage, inflammation, and insulin resistance. Enhancing FAO *via* LCA attenuated lipotoxicity and lipotoxicity-induced pathological changes in the atria of obese mice, resulting in restored structural remodeling and ameliorated AF susceptibility. Mechanistically, LCA activated AMPK/PGC1α signaling both *in vivo* and *in vitro*, and pharmacological inhibition of AMPK *via* Compound C attenuated LCA-induced cardio-protection in palmitate-treated primary atrial cardiomyocytes. Taken together, our results demonstrated that FAO promotion *via* LCA attenuated obesity-mediated AF and structural remodeling by activating AMPK signaling and alleviating atrial lipotoxicity. Thus, enhancing FAO may be a potential therapeutic target for AF.

## Introduction

Atrial fibrillation (AF), the most common sustained cardiac arrhythmia in clinical practice affecting nearly 2% of general population, is associated with substantial complications and financial burden (Lippi et al., 2021). Metabolic disturbances have shown strong relationship with AF by ample clinical evidence, and have been represented as driving forces for adverse atrial remodelling mechanically (Mourtzinis et al., 2018). In addition, regarding cardiac high energy demand, disordered atrial metabolism is supposed to take a leading role in AF pathogenesis. However, far less is known about the impacts of atrial metabolism in AF.

Metabolic homeostasis and abnormalities have spurred major interest in the field of AF at present, with a focus on lipids, the predominant energy substrates (∼70%) of heart. Under physiological conditions, absorbed cardiac FAs is delivered into mitochondria *via* the gateway enzyme carnitine palmitoyltransferase-1B (CPT1B), and fueled by mitochondrial FAs oxidation (FAO) and the TCA cycle. In context of AF, the metabolic disturbance of FAs was observed and proved to contribute to the predisposition and perpetuation of AF (Mourtzinis et al., 2018). Briefly, during AF, irregular high-frequency excitation and contraction of cardiomyocytes shift the metabolic balance from FAO to carbohydrate utilization, a more oxygen-saving way (Heijman and Dobrev, 2015). Supportively, AF patients and animals show the coordinated transcriptional down-regulation of FAO-related enzymes (especially AMP-activated protein kinase (AMPK), peroxisome proliferator-activated receptor γ coactivator1α (PGC1α), and CPT1B) and concomitant up-regulation of glycolysis-related enzymes in atria (Barth et al., 2005; Tu et al., 2014; Mourtzinis et al., 2018; Jie et al., 2019). Supportively, redressing lipid metabolism through factors, such as AMPK, PPARα/PGC1α, Hif1-α, VLCAD, and PLIN2, has been proved effective to prevent AF, exemplifying metabolic modulation as a potential therapeutic strategy for AF (Harada et al., 2017). Of note, pharmacological interventions targeting FAO regulators, AMPK and PGC1α (Yu et al., 2011; Liu et al., 2016; Bai et al., 2019; Deshmukh et al., 2021; Ostropolets et al., 2021), have been proved to reduce AF susceptibility, yet it is still unclear whether enhancing FAO alleviates AF.

AF risk escalates in parallel with increased BMI, thus obesity, a public health issue as well as the most common metabolic disorder in human, is regarded as the second biggest attributable risk factor for AF (Wong et al., 2015). Various pathological conditions including metabolic imbalance of glucose or lipid and metabolic stress has been observed in obesity (Vyas and Lambiase, 2019). Notably, diet-induced obesity increases the influx of FAs and downregulates the key enzymes involved in FAs expenditure (Haffar et al., 2015; Heier and Haemmerle, 2016; Garcia and Shaw, 2017). Particularly, in long-term obese individuals, CPT1B is decreased in expression and blunted in response to lipid (Maples et al., 2015), while L-carnitine (LCA), the obligatory cofactor of CPT1B (Söder et al., 2019), is decreased in serum and insufficient to cope with FAs overload (Pooyandjoo et al., 2016). Together, obesity and AF share the same pathogenesis, defective FAO, which might count for the increased AF susceptibility, especially in which are provoked by obesity. Therefore, FAO promotion maybe the first-line option to combat AF, especially obesity-related AF. In pathological context, ectopic lipid accumulation and consequently lipotoxicity occurred when FAO was defective in cardiomyocyte, serving as a mechanistic link between AF/obesity and metabolic disorder (Haffar et al., 2015; Ozcan et al., 2015; Opacic et al., 2016; Harada et al., 2017). Specifically, cardiac lipotoxicity could provoke oxidative stress, DNA damage, inflammation, and insulin intolerance, contributing morphological changes and cellular dysfunction of atria (Karam et al., 2017; Sletten et al., 2018), including cardiac hypertrophy, fibrosis, gap junction remodeling, and myocardial injury (Shenasa et al., 2015; Fukui et al., 2017; Meng et al., 2017; Sato et al., 2019), thus, providing substrates for AF.

Therefore, we adopted LCA (150 mg/kg⋅BW/d) to facilitate FAO, and further examined the AF vulnerability and atrial remodeling *in vivo* (a mice model of high-fat diet (HFD)-induced obesity-mediated AF) and *in vitro* (a primary atrial cardiomyocyte cell model of palmitate (PA)-mimicked lipid overloading), aiming to determine whether enhancing FAO can alter the process that underlie AF in obesity and explore possible mechanisms. Our results determined that defective cardiac FAO takes a leading role in obesity-related AF, and proved that FAO promotion *via* LCA exerts an anti-AF effect through activating AMPK signals and reducing atrial lipotoxicity in obese mice. Notably, this article firstly demonstrates the beneficial effects of enhanced FAO in the reversal of obesity-related AF, thus shedding light onto a feasible AF treatment.

## Methods and Materials

### Animal and Treatment

Male C57BL/6J mice (aged 4–6 weeks) were purchased from Xi’an Jiaotong University (Xi’an, China) and bred under standard laboratory conditions. After 1 week of acclimatization, a total of forty mice were randomly divided into 4 groups (*n* = 10 per group): 1) Standard diet group (STD; 20% fat, 56% carbohydrate, 24% protein; Research Diets Inc., New Brunswick, NJ); 2) STD + LCA group; 3) HFD group (HFD; 60% fat, 20% carbohydrate, 20% protein; Research Diets Inc.); 4) HFD + LCA group. After feeding HFD for 8 weeks, the mice became obese and showed greater propensity for AF. Subsequently, FAO activator, LCA (150 mg/kg⋅BW/d; TargetMol, Boston, United States) (Bakermans et al., 2013), was administrated *via* drinking water for another 4 weeks. Based on our preliminary experiment, the experimental dose of LCA was set to be 150 mg/kg⋅BW/d, which had no significant impact on mice body weight (BW) but suppressed obesity-induced AF susceptibility. At the end of the experiment, AF induction, echocardiography, intraperitoneal glucose tolerance test (IPGTT) and insulin tolerance test (ITT) were performed in each group before tissue sampling. After overnight fasting, atrial tissues and blood samples were collected from euthanized mice for further analysis. All the procedures of this study were approved by the Institutional Animal Care and Use Committee of Xi’an Jiaotong University.

### Cell Culture and Treatment

Primary atrial cardiomyocytes were isolated from the atria of neonatal Sprague–Dawley rat (1∼3-day-old; Xi’an Jiaotong University, Xi’an, China). Briefly, atria tissue were surgically removed, trypsinized (0.08% trypsin; Solarbio, Beijing, China), digested with 0.1% collagenase Ⅱ (Solarbio), and the primary atrial cardiomyocytes were isolated by differential detachment and verified under the microscope. Isolated cells were cultured in 6-well plates at 37°C in 5% CO_2_, with DMEM supplemented with 10% FBS and 1% penicillin/streptomycin solution. Final solutions of 200 μM PA (dissolved in 20% BSA; Sigma-Aldrich, St. Louis, United States) was added to replicate the effects of lipid overloading, while LCA (5 mM; TargetMol) was added to enhance FAO, and Compound C (CC, 0.5 μM; Sigma-Aldrich) was added to inhibit AMPK activation. After being treated for 24 h, cells were collected, washed and lysed for the following evaluations.

### AF Induction and Electrophysiological Examination

Programmed *trans*-esophageal stimulation was performed to assess AF inducibility as described previously (Fu et al., 2021). Briefly, mice were anesthetized with pentobarbital intraperitoneally (50 mg/kg⋅BW) and then electrical stimulated by an external simulator (SCOPE, Kaifeng, China), and surface electrocardiogram (ECG) was recorded by a physiologic signal-acquisition system (RM6240; Chengdu instrument factory, Chengdu, China). At first, baseline ECG was analyzed by 10 consecutive beats recorded in the initial stabilization period with heart rates between 300 and 500 bpm at first. Later, AF was induced with burst pacing (pulse width 1 ms; 1.5× capture threshold; 30, 35, and 40 Hz), and was considered sustained as persisted rapid irregular f-waves with irregular R-R intervals lasting for more than 1 s. In addition, atrial effective refractory period (ERP) was assessed by continuous stimulation applied with decreasing R-R intervals from 140 to 40 ms at 1 ms decrements. Sinoatrial node recovery time (SNRT_max_) was measured as the longest duration between the last stimulus and the first sinus P-wave, and corrected by the R-R interval (_c_SNRT_max_).

### Echocardiography

2D echocardiography was employed to discern cardiac structural and functional differences among groups. Echocardiography (Vevo 2,100; VisualSonics Inc., Toronto, Ontario, Canada) was performed by an animal cardiologist blind to the experimental design in mice anaesthetized with inhalational isoflurane. Dimension of superoinferior (SI), anteroposterior (AP) and mediolateral (ML) were obtained in a long-axis view and a short-axis view, respectively. LA filling volume was calculated using the formula: LA Volume = (4π×SI×AP×ML)/(3 × 2×2 × 2) (Ujino et al., 2006). Cardiac function was calculated according to standard formulae, and the results were averaged of three cardiac cycles.

### IPGTT and ITT

Glucose and insulin homeostasis were evaluated *in vivo* by IPGTT and ITT. For the IPGTT, D-glucose was injected intraperitoneally (2 g/kg⋅BW) into over-night fasted mice, and, subsequently, blood was taken from the tail vein at 5, 15, 30, 60, 90 and 120 min after glucose loading and blood glucose levels were measured by the Accu-Chek glucometer (Roche Diagnostics, Indianapolis, United States). Similarly, for the ITT, mice were fasted for 2 h before insulin administration intraperitoneally (1 U/kg⋅BW; Wanbang Biopharma, Xuzhou, China), following the glucose determination at 0, 15, 30, 45 and 60 min. Glucose tolerance and insulin sensitivity were calculated as the area under the curve (AUC) by GraphPad Prism 8.0 (GraphPad Software, San Diego, America).

### Analysis of Histological Staining and Fluorescence

After being isolated from heart tissue, atrial appendages were fixed with 4% paraformaldehyde overnight, embedded in paraffin, and cut into 5 µm slides longitudinally. Paraffin-embedded specimens were finally stained with hematoxylin-eosin (H&E), Wheat Germ Agglutinin (WGA), Masson, Periodic acid-Schiffs (PAS), selected antibodies (connexin-43; 1:200; Invitrogen, California, United States, NRF2; 1:500; Proteintech, Chicago, United States, and 8-Hydroxy-2′-deoxyguanosine (8-OH-dG), 1: 100; JalCA, Shizuoka, Japan), and TUNEL (*in situ* cell death detection kit, TMR red; Roche Diagnostics). Respectively, frozen sections (10 µm thickness) of atria samples were prepared and stained with oil red O, MitoSOX and dihydroethidium (DHE). Slides were visualized and photographed by an optical microscope (Nikon, Melville, NY, United States) at a magnification of 100× for histology, or by a confocal microscope (Leica, Bensheim, Germany) at a magnification of 20× for immunofluorescence. Images were further analyzed by ImageJ software (version 1.46r; National Institutes of Health, Bethesda, United States). Data were expressed as the percentage of the positive-stained region to the total area of cardiomyocyte area, and averaged from six random fields in each slide.

### Detection of Markers in Serum and Tissue

Biomarkers were assayed using commercially available kits in accordance with manufacturer’s guidelines, including fasted non-esterified FAs (NEFA) (Solarbio), lactate dehydrogenase (LDH) (Nanjing Jiancheng Bioengineering Institute, Nanjing, China), creatine kinase-MB (CK-MB) (Nanjing Jiancheng Bioengineering Institute), malondialdehyde (MDA) (Beyotime Institute of Biotechnology, Shanghai, China) and superoxide dismutase (SOD) (Beyotime Institute of Biotechnology).

### Assay of FAO *in vitro*

Cellular FAO was measured with a FAO Assay Kit (ab222944; Abcam, Cambridge, United States) according to the manufacturer’s instruction. Fluorescence was detected using FLUOstar Omega (BMG LABTECH, Aylesbury, United Kingdom) and the results were normalized by protein concentration in each well through a micro-bicinchoninic acid (BCA; Pierce Chemical Company, Rockford, United States) kit.

### Western Blot

Protein levels were measured by WB with β-actin as the loading control. Protein was extracted from atria samples or cells and quantitated by a BCA assay. Equal amount (10–30 μg) of protein was loaded and separated by SDS-PAGE using 10 or 12% acrylamide gradients, and later transferred to nitrocellulose membranes. The membranes were blocked with 5% skim milk and incubated with antibodies against total-AMPK (1:1,000; Cell Signaling Technology/CST, Massachusetts, United States), CD36 (1:1,000; Abcam), collagen Ⅰ (1:1,000; Abcam), collagen Ⅲ (1:1,000; Abcam), connexin-43 (1:200; Invitrogen), CPT1B (1:1,000; Proteintech), GLUT4 (1:500; Proteintech), NFκB (1:1,000; Proteintech), NRF2 (1:1,000; Proteintech), phoso-AMPK (Thr172; 1:1,000; CST), phoso-Akt (Ser473; 1:1,000; CST), phoso-NFκB (1:1,000; CST), PGC1α (1:1,000; Proteintech), SOD2 (1:1,000; Proteintech), TGF-β (1:1,000; Abcam), α-SMA (1:1,000; CST), β-actin (1:5,000; Proteintech). Protein levels were quantified as the intensity of bands using ImageJ software (NIH systems) and standardized to β-actin. Abbreviations are fully illustrated in corresponding figure legends.

### RNA Extraction and Real-Time Reverse Transcription Polymerase Chain Reaction

RT-qPCR was carried out to quantify gene expressions among the groups. Briefly, RNA from atria tissues was extracted using TRIzol reagent (Accurate Biology, Changsha, China). Then, the high-sensitivity RT-qPCR reaction was measured using the SYBR green chimeric fluorescence method (Accurate Biology) and detected by CFX96™ Real-Time PCR System (Bio-Rad, Hercules, CA, United States). Results were quantified using the 2^-△△Ct^ comparative method and normalized by β-actin. The primer sequences and full names of genes are listed in Supplementary Table S1.

### Statistical Analysis

All data of animal and cell studies were analyzed by SPSS 23.0 (IBM SPSS software, New York, United States), visualized by GraphPad Prism 8.0 (GraphPad Software), and shown as mean ± SEM. Replicates are indicated in figure legends and table legends. Data normality was evaluated by Kolmogorov-Smirnov test, and comparisons between groups were performed with One-way ANOVA (in mice experiments) or Two-way ANOVA (in cell experiments) with Bonferroni *post-hoc* test. For all tests, *p* < 0.05 was considered significant.

## Results

### LCA Supplementation Attenuates Obesity-Induced AF Susceptibility

**FIGURE 1:**
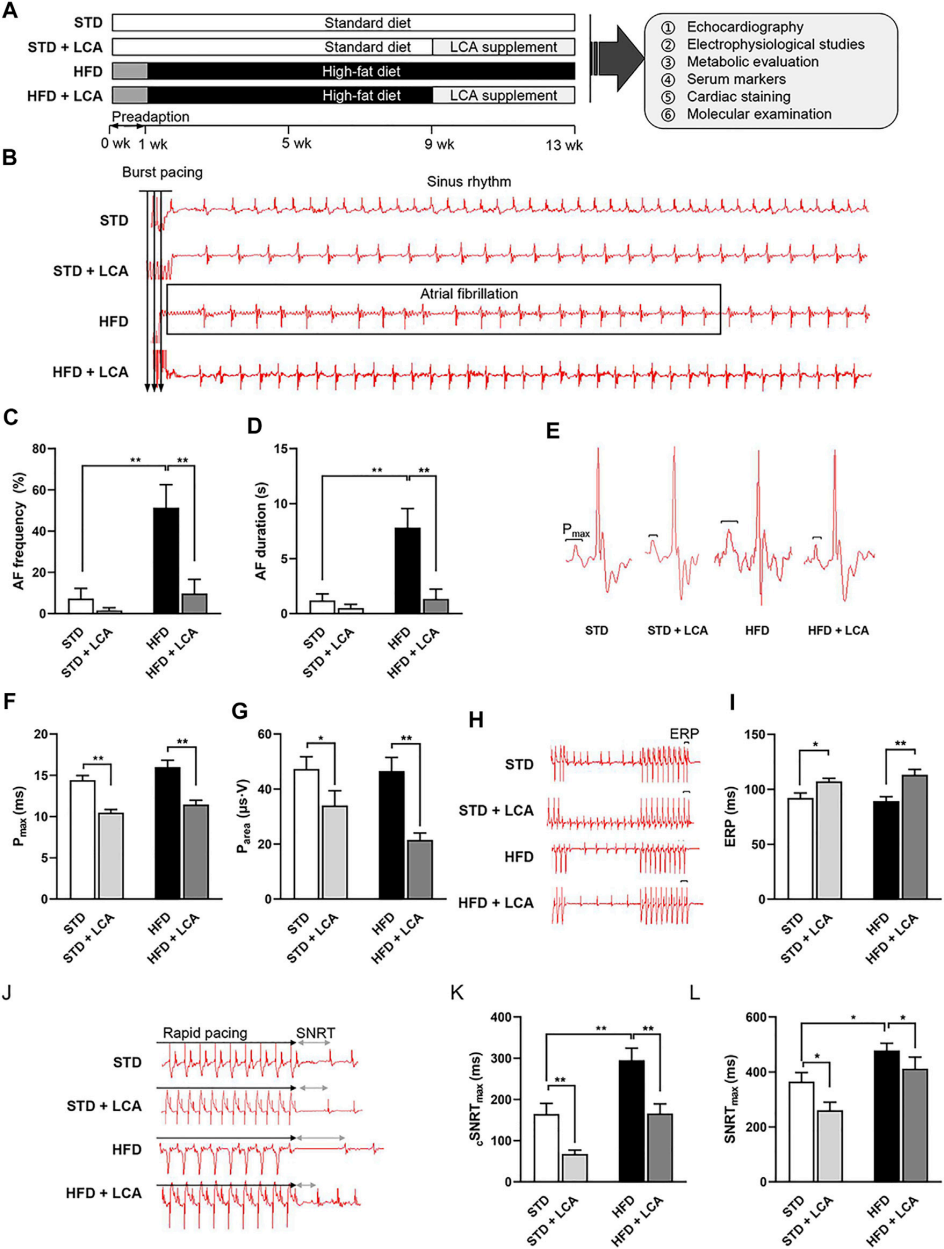
LCA inhibits obesity–induced AF. **(A)** Experimental protocols of this study. **(B)** Representative AF induction by *trans*-esophageal burst pacing. **(C,D)** Analysis of AF frequency and duration. **(E)** Three-limb-lead electrocardiogram at baseline. **(F,G)** Analysis of P-wave. **(H–L)** Analysis of SNRT_max_, _c_SNRT_max_ and ERP detected by programmed cardiac stimulation. *n* = 10 per group. One-way ANOVA with Bonferroni *post-hoc test* was used to compare data among groups. Data are expressed as mean ± SEM. ^*^ *p* < 0.05, ^**^ *p* < 0.01. STD, standard diet; HFD, high-fat diet; LCA, l-carnitine; FAO, fatty acids oxidation; P_area,_ P-wave area; P _max_, P-wave duration; AF, atrial fibrillation; ERP, effective refractory period; SNRT, sinus node recovery time; _c_SNRT, corrected sinus node recovery time.

In this study, we established a mouse model of obesity-related AF through 13-week HFD feeding, and LCA was administrated *via* drinking water (150 mg/kg⋅BW/d) during the last 4-week period to investigate whether enhancing FAO could attenuate obesity-mediated AF. The schematic of the experimental design is shown in Figure 1A, and basal characteristics of mice in each group are summarized in Table 1 *.*

**TABLE 1:** Physical characteristics, echocardiographic, surface ECG and electrophysiological parameters, and serum marker levels of STD, STD + LCA, HFD and HFD + LCA mice.

Parameter	STD	STD + LCA	HFD	HFD + LCA
	(*n* = 10)	(*n* = 10)	(*n* = 10)	(*n* = 10)
Physical characteristics				
Food intake (g/d)	3.39 ± 0.18	3.36 ± 0.13	2.48 ± 0.06^*^	2.53 ± 0.18^*^
Energy intake (kcal/gm/d)	13.02	12.90	12.97	13.27
Water intake (ml/d)	5.12 ± 0.09	5.14 ± 0.13	4.82 ± 0.05^*^	4.83 ± 0.16
Body weight (g)	26.21 ± 0.69	27.17 ± 0.96	33.41 ± 0.55^*^	35.91 ± 1.24
Heart weight (mg)	132.10 ± 10.59	129.33 ± 3.83	155.70 ± 7.83^*^	143.50 ± 3.37^†^
NAL (cm)	10.04 ± 0.09	10.00 ± 0.04	10.17 ± 0.08	10.09 ± 0.11
HW/NAL (mg/cm)	13.16 ± 0.53	12.18 ± 0.53	15.32 ± 0.46*	14.01 ± 0.09^†^
Heart rate (bpm)	378.80 ± 20.64	364.70 ± 15.43	414.70 ± 22.12	386.1 ± 34.78
Echocardiography				
LA diameter (mm)	1.86 ± 0.07	1.61 ± 0.07	2.39 ± 0.09*	1.97 ± 0.12^†^
LA filling volume (ml)	23.26 ± 4.40	18.38 ± 3.26	58.68 ± 15.50*	21.26 ± 5.71^†^
Surface ECG				
P _max_ (ms)	14.43 ± 0.56	10.47 ± 0.39*	16.00 ± 0.83	11.45 ± 0.53^†^
P_area_ (μs·V)	47.32 ± 4.49	36.23 ± 2.74*	46.57 ± 4.95	21.52 ± 2.50^†^
Electrophysiology				
AF duration (s)	1.08 ± 7.00	0.50 ± 0.34	154.77 ± 49.33*	18.69 ± 14.63^†^
AF frequency (%)	9.70 ± 6.95	1.60 ± 1.30	51.40 ± 11.10*	7.30 ± 4.96^†^
AF incidence (%)	30	20	90.00*	30.00^†^
SNRT _max_ (ms)	364.90 ± 33.07	261.10 ± 28.51*	478.40 ± 26.47*	412.20 ± 41.51^†^
_c_SNRT _max_ (ms)	164.80 ± 25.75	67.90 ± 90.4*	295.60 ± 29.08*	166.00 ± 23.24^†^
ERP (ms)	92.20 ± 4.57	107.20 ± 2.88*	89.40 ± 4.07	113.20 ± 4.97*^†^
Serum markers				
NEFA (mmol/L)	0.88 ± 0.18	0.76 ± 0.10	1.39 ± 0.16*	0.65 ± 0.11^†^
LDH (U/L)	57.85 ± 7.97	60.19 ± 8.26	90.38 ± 7.95*	75.97 ± 6.79
CK-MB (U/L)	126.00 ± 7.77	134.69 ± 15.33	137.69 ± 2.41	81.31 ± 26.68^†^
MDA (μmol/L)	0.78 ± 0.13	0.79 ± 0.37	1.67 ± 0.22^*^	0.53 ± 0.07^†^
SOD (U/ml)	34.55 ± 3.53	39.19 ± 2.03	32.18 ± 2.38	41.16 ± 6.37*^†^
Heart markers				
MDA (μmol/mg prot)	0.53 ± 0.03	0.43 ± 0.11	1.26 ± 0.02*	0.38 ± 0.09^†^
SOD (U/mg prot)	602.25 ± 42.36	677.25 ± 57.79^*^	417.19 ± 45.15*	838.61 ± 111.19^†^

*n* = 10 per group. One-way ANOVA, with Bonferroni *post-hoc* test was used to compare data among groups. The data were expressed as mean ± SEM. ^*^ *p* < 0.05 vs STD, ^†^ *p* < 0.05 vs HFD. AF, atrial fibrillation; _c_SNRT, corrected sinus node recovery time; ECG, electrocardiogram; ERP, effective refractory period; HFD, high-fat diet; HW, heart weight; LA, left atrium; LCA, l-carnitine; LDH, lactate dehydrogenase; CK-MB, creatine kinase-MB; MDA, molondialdehyde; NAL, naso-anal length; NEFA, non-esterified fatty acids; P_area,_ P-wave area; P _max_, P-wave duration; SNRT, sinus node recovery time; SOD, superoxide dismutase; STD, standard diet.

As expected, at the end of the experiment, HFD mice gained 20% more BW than STD mice (Supplementary Figure S1A), together with increased heart weight (HW) and serum NEFA (Supplementary Figures S1B–D). Obesity-mediated AF was successfully established, as supported by increased AF frequency and prolonged duration in HFD mice (Figures 1B–D). LCA decreased the ratio of HW/NAL (nasal-anal length) in HFD mice, yet no significant change of BW gain was observed with or without LCA supplementation (Supplementary Figures S1A–C). In addition, LCA suppressed HFD-induced elevation of serum NEFA (Supplementary Figure S1D). Of note, LCA supplementation alleviated pacing-induced AF susceptibility in the obese mice. Surface ECG (Lead II) at baseline showed that LCA supplementation significantly decreased P-wave duration (P_max_) and P-wave area (P_area_), two of the independent predictors for AF, in obese mice (Figures 1E–G). In addition, electrophysiological abnormalities, including prolonged SNRT_max_ and shortened ERP, were regarded to increase the AF propensity. Supportively, LCA supplementation shortened SNRT_max_ and extended ERP in obese mice (Figures 1H–L).

### LCA Supplementation Constrains Obesity-Induced Atrial Structural Remodeling

Atrial structural remodeling, including atrial dilatation, cellular hypertrophy, gap junction disturbance, and interstitial fibrosis, offers substrates for AF. Thus, we later assessed atrial structural alterations among the groups.

**FIGURE 2:**
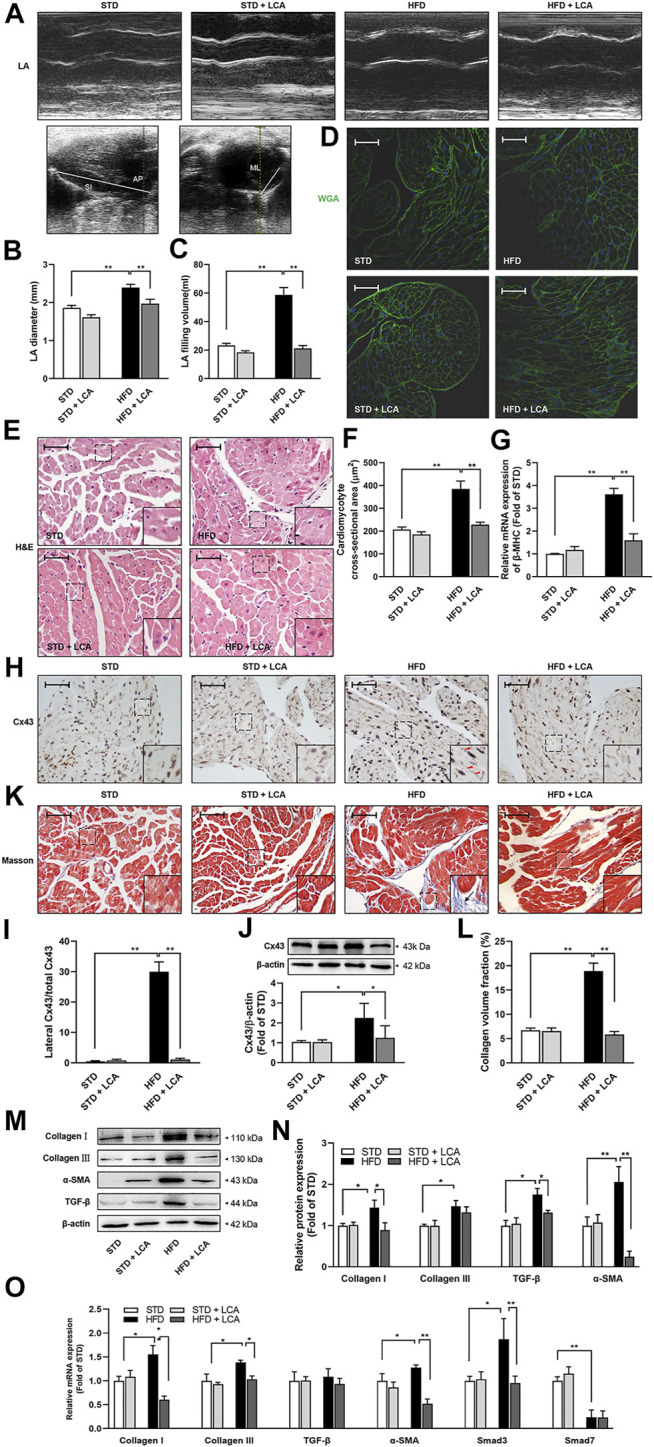
LCA suppresses atrial remodeling in obese mice. **(A)** Representative echocardiographic images of LA among the groups. **(B)** Measurements of LA diameter and **(C)** LA filling volume detected by 2D-guided M-mode imaging. The SI and AP were obtained in a long-axis view, and ML was assessed in parasternal short-axis view. LA filling volume was calculated using the formula: LA filling volume = (4π×SI×AP×ML)/(3 × 2× 2 × 2). **(D)** Representative sections of WGA staining. **(E)** Representative sections of H&E staining. **(F)** Quantitative analysis of cellular morphology by ImageJ. **(G)** Relative mRNA levels of β-MHC using RT-qPCR. **(H,I)** Subcellular localization of Cx43 and immunohistochemistry. Triangles indicate lateralized Cx43. **(J)** Protein expression of Cx43 using Western blot. **(K,L)** Interstitial fibrosis (Arrow) using Masson’s trichrome staining. **(M,N)** Representative images and analysis of TGF-β signaling-associated proteins (TGF-β, α-SMA, collagen Ⅰ and collagen Ⅲ) using Western blot. **(O)** Relative mRNA expression levels of the fibrosis-related genes (TGF-β, α-SMA, Smad3, Smad7, collagen Ⅰ and collagen Ⅲ) using RT-qPCR. Scale bar: 50 μm *n* = 10 **(A–C)** or 4 **(D–O)** per group. One-way ANOVA with Bonferroni *post-hoc* test was used to compare data among groups. Data are expressed as mean ± SEM. ^*^ *p* < 0.05, ^**^ *p* < 0.01. STD, standard diet; HFD, high-fat diet; LCA, l-carnitine; FAO, fatty acids oxidation; LA, left atrium; SI, superoinferior dimension; AP, anteroposterior dimension; MI, mediolateral dimension; WGA, wheat Germ Agglutinin; H&E, hematoxylin-eosin; β-MHC, β–cardiac myosin heavy chain; Cx43, connexin-43; TGF-β, transform growth factor-β; α-SMA, α-smooth muscle actin; Smad, *drosophila* mothers against decapentaplegic protein.

Obese mice displayed excessive left atrial enlargement, as shown by marked increase of left atrium (LA) diameter and LA filling volume (Figures 2A–C). In atrial cardiomyocytes, obesity developed substantial cellular hypertrophy and disarray in the atria, as evidenced by H&E staining, WGA staining and β-MHC transcription (Figures 2D–G). Moreover, the main gap junction protein, connexin-43, was apparently upregulated in expression and heterogeneous distributed in the atrial cardiomyocyte of obese mice (Figures 2H–J). In addition, intra-myocardial fibrosis, a hallmark of AF, was observed to increase in the atria of obese mice, as evidenced by aggravated collagen deposition, upregulated pro-fibrosis signaling (TGF-β, α-SMA, Smad3, collagen Ⅰ, and collagen Ⅲ), and downregulated transcription of anti-fibrotic Smad7 (Figures 2K–O).

Coincided with reduced AF inducibility, LCA supplementation showed significant capacity to shrink LA, attenuate cellular hypertrophy, gap junction disturbance, and fibrosis while challenged by a long-term HFD (Figure 2), indicating FAO promotion *via* LCA supplementation constrained the AF substrates in obese mice.

### Enhanced FAO *via* LCA Supplementation Redresses Lipid Metabolism Imbalance, Thereby Decreasing Obesity-Induced Lipid Deposition in Atria

**FIGURE 3:**
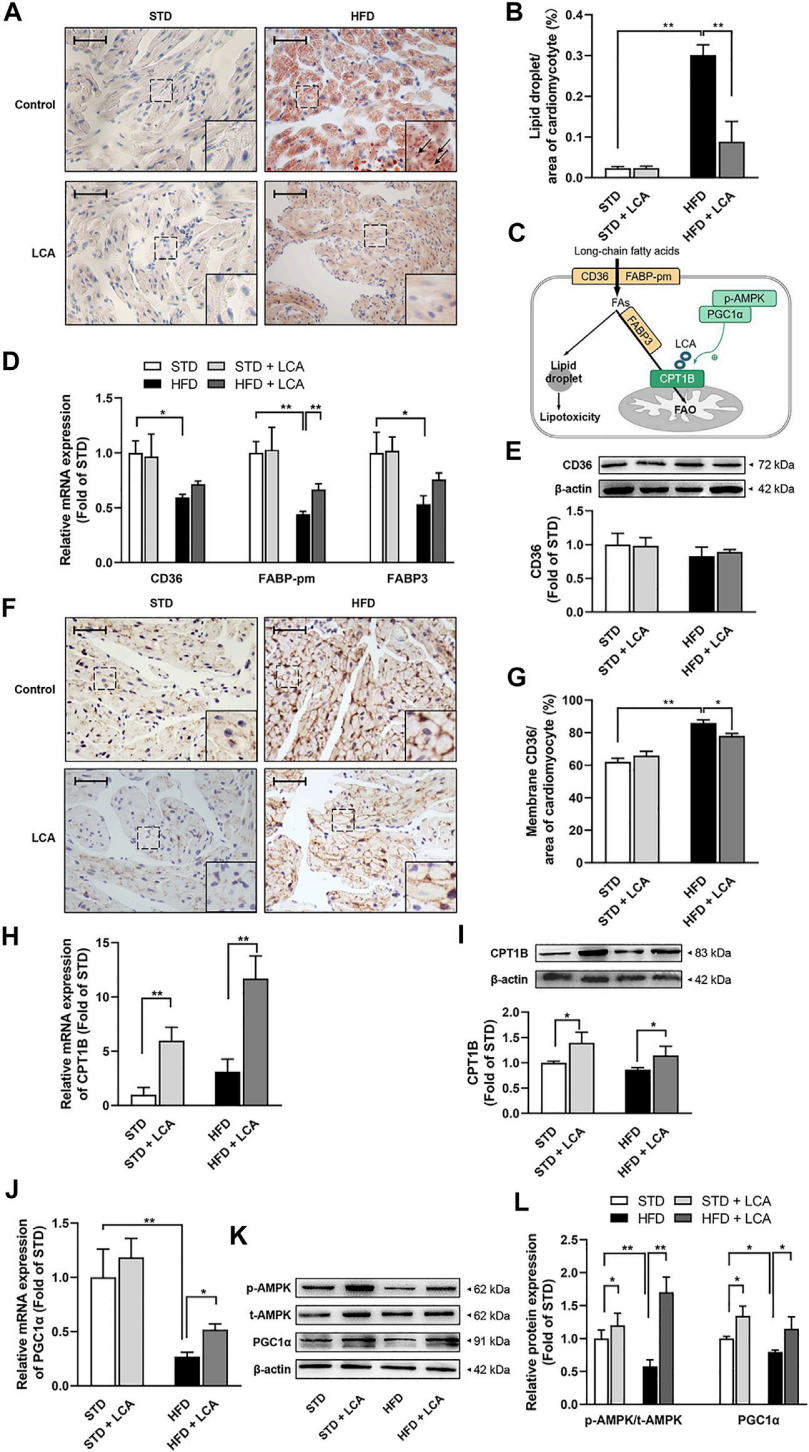
LCA enhances FAO, thereby inhibits cardiac lipotoxicity by alleviating atrial steatosis in obese mice. **(A)** Representative sections of lipid accumulation (Arrow) using oil red O staining. **(B)** Quantitative analysis of lipid accumulation by ImageJ. **(C)** Schematic diagram illustrating cardiac lipid metabolism. **(D)** Quantitative analysis of the transcription of lipid uptake and transportation-related genes (CD36, FABP-pm, FABP3) by RT-PCR. **(E)** Representative image and quantitative analysis of CD36 by Western blot. **(F)** Representative images of membrane translocation of CD36 using immunohistochemistry. **(G)** Quantitative analysis of sarcolemma CD36 contents by ImageJ. **(H)** Quantitative analysis of the transcription of CPT1B using RT-qPCR. **(I)** Representative Image and quantitative analysis of CPT1B by Western blot. **(J)** Quantitative analysis of the transcription of PGC1α using RT-qPCR. **(K,L)** Representative images and quantitative analysis of FAO-related regulators (AMPK and PGC1α) by Western blot. Scale bar: 50 μm *n* = 4 per group. One-way ANOVA with Bonferroni *post-hoc* test was used to compare data among groups. Data are expressed as mean ± SEM. ^*^ *p* < 0.05, ^**^ *p* < 0.01. STD, standard diet; HFD, high-fat diet; LCA, l-carnitine; FAs, fatty acids; FAO, fatty acids oxidation; AMPK, AMP-activated protein kinase; CD36, FAT; CPT1B, carnitine palmitoyltransferase-1B; PGC1α, peroxisome proliferator-activated receptor γ coactivator1α; FABP-pm, plasma membrane fatty acid-binding protein; FABP3, fatty acid binding protein 3; p-, phoso-.

Epigenetic studies demonstrated that AF is highly associated with lipid metabolic abnormalities. In line with this, apparent lipid deposition was observed in obese mice. What’s more, in accordance with reduced AF susceptibility and atrial remodeling, promoting FAO with LCA decreased the atrial lipid deposition effectively in obese mice (Figures 3A,B).

Promoted FAs influx and defective FAO can predispose to lipid deposition (Figure 3C). Thus, firstly, we assessed factors involved in lipid uptake and transportation. CD36, the main *trans*-membrane translocase of FAs, was decreased in gene level in obesity, and increased after LCA treatment (Figure 3D). However, CD36 was unchanged in total protein level among the groups (Figure 3E), but elevated in atrial membrane translocation in obese mice, and slightly reduced after LCA supplementation (Figures 3F,G). Besides, the transcription of other transportation-related genes, FABP3 and FABP-pm, was decreased in the atria of obesity and restored after LCA supplementation (Figure 3D).

Next, we evaluated the expression of the key enzymes of FAO, including CPT1B, AMPK and PGC1α, by RT-qPCR and Western blot. In contrast to increased FAs uptake, mitochondrial FAO was downregulated in obesity, as evidenced by the remarkable decrease of AMPK phosphorylation, and PGC1α expression in the atria of HFD mice compared to STD mice (Figures 3H–L). Notably, LCA supplementation upregulated the phosphorylation of AMPK as well as the expression of PGC1α and CPT1B in HFD mice (Figures 3D–L).

### LCA Supplementation Restrains Cardiac Oxidative Stress and DNA Damage in the Atria of Obese Mice

**FIGURE 4:**
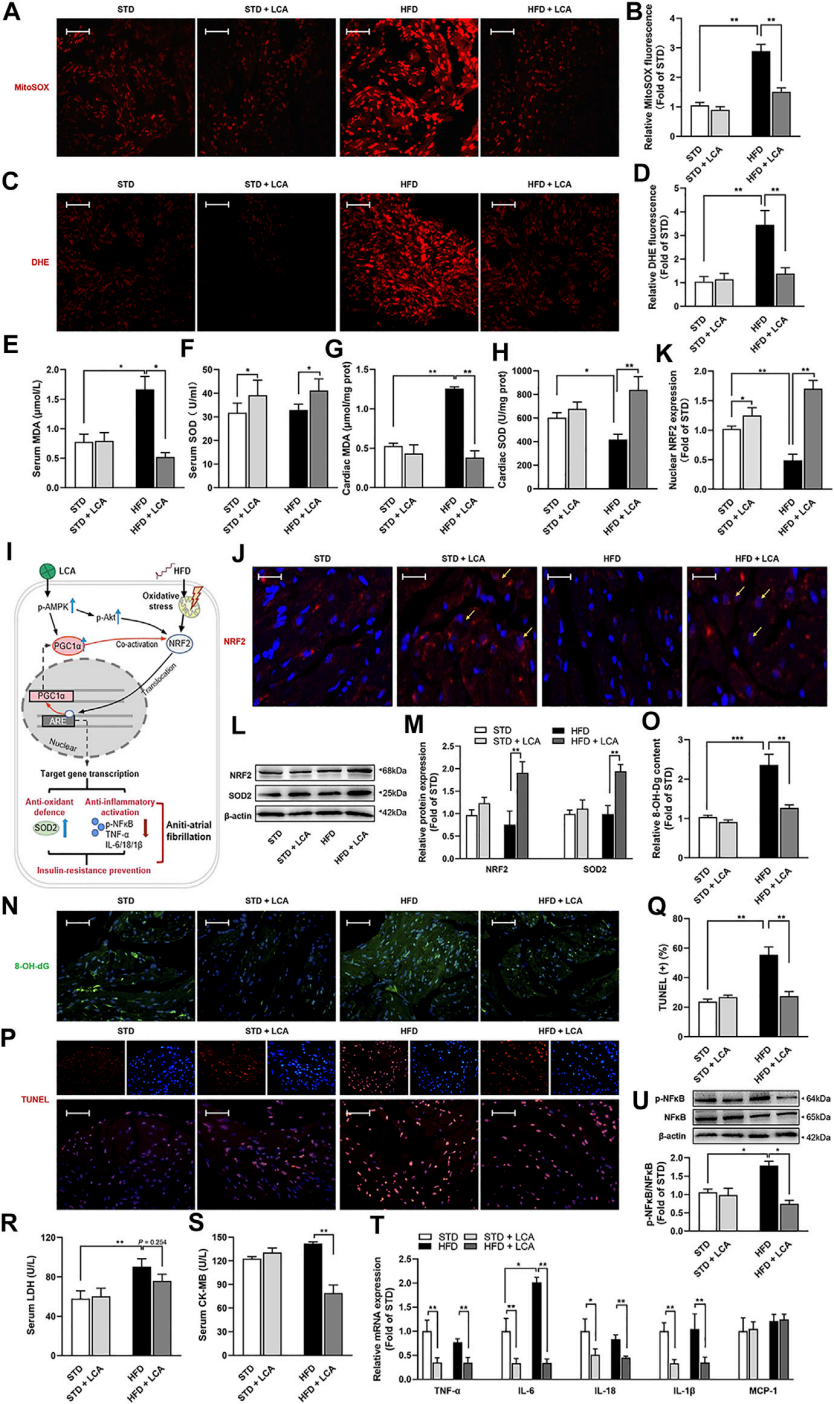
LCA inhibits cardiac oxidative stress and mitigates inflammation. Representative images and analysis of the subcellular localization of the oxidation products of **(A,B)** MitoSOX and **(C,D)** DHE. Red: oxidation products-staining. Scar: 10 μm *n* = 4. **(E,F)** Comparisons of serum MDA and SOD using commercially available kits among the groups. **(G,H)** Levels of atrial MDA and SOD normalized to tissue protein concentration. **(I)** Schematic diagram illustrating NRF2-related signals. **(J,K)** Localization of NRF2 in atria by confocal immune-cyto-chemical analysis: Blue: nucleus (DAPI); Red: NRF2-staining; Pink: merge of blue and red indicated nuclear localization of NRF2 (Arrow). Scar: 30 μm *n* = 4. **(L,M)** Representative images and quantitative analysis of anti-oxidative system involved protein expressions (NRF2 and SOD2) using Western blot. **(N,O)** Representative images and analysis of oxidative DNA damage by 8-OH-dG staining. Green: 8-OH-dG -staining; Blue: DAPI. Scar: 50 μm *n* = 4. **(P,Q)** Representative images and analysis of DNA damage by TUNEL staining. Red: TUNEL-staining; Blue: DAPI; Pink: merge. Scar: 50 μm *n* = 4. Comparisons of **(R)** serum LDH and serum **(S)** CK-MB using commercially available kits. *n* = 8. **(T)** Quantitative analysis of the expression of inflammation-related genes in the atria using RT-qPCR. **(U)** Representative images and quantitative analysis of expression and phosphorylation of NFκB using Western blot. One-way ANOVA with Bonferroni *post-hoc* test was used to compare data among groups. Data are expressed as mean ± SEM. ^*^ *p* < 0.05, ^**^ *p* < 0.01. STD, standard diet; HFD, high-fat diet; LCA, l-carnitine; DHE, dihydroethidium; MDA, molondialdehyde; SOD, superoxide dismutase; NRF2, nuclear erythroid 2 p45-related factor 2; p-, phoso-; SOD2, manganese superoxide dismutase, superoxide dismutase 2; 8-OH-Dg, 8-hydroxydeoxyguanosine; IL, interleukin; TNF-α, tumor necrosis factor-α; MCP-1, monocyte chemoattractant protein-1; NFκB, the nuclear factor kappa B; LDH, lactate dehydrogenase; CK-MB, creatine kinase-MB.

Lipid over-deposition undermines the structure of cardiomyocytes, namely, cardiac lipotoxicity (Sletten et al., 2018), thereby provoking a set of pathological processes, including oxidative inflammation, DNA damage and insulin resistance, which have been implicated as possible mechanisms for AF. Thus, firstly, we evaluated oxidative stress. Obesity increased mitochondrial superoxide production (measured by MitoSOX staining) and increased cellular oxidative stress (measured by DHE staining) in the atria (Figures 4A–D). Besides, obesity increased MDA contents in the serum and atria, and decreased atrial SOD, but unaffected serum SOD level. LCA supplementation thwarted oxidative damages provoked by HFD, as supported by the decreased MitoSOX Red and DHE fluorescence signal, the reduced MDA levels and the rise of SOD levels in the serum and atria of obese mice (Figures 4A–H).

Nuclear factor erythroid 2-related factor (NRF2) is an accepted master regulator participating in the cellular adaptive response to redox or energy stress, which accumulates in nuclear to induce a battery of defensive genes encoding detoxifying enzymes and antioxidant proteins (such as SOD2 and NFκB) (Figure 4E) (Tonelli et al., 2018). Next, we evaluated the NRF2-mediated cardio-protective pathways. Nuclear levels of NRF2 were comparably decreased in the atria of obese mice, and increased after LCA supplementation (Figures 4J,K). In addition, total protein level of NRF2 was elevated after LCA supplementation. In parallel with the distinct NRF2 activation, SOD2, one of the downstream of NRF2 for antioxidant defense, was noticeably increased in the atria of obese mice after LCA treatment (Figures 4L,M).

Consistent with obesity-induced oxidative stress, oxidative DNA damage (as shown by 8-OH-dG staining) and DNA segment (as shown by TUNEL staining) in the atira was aggravated after HFD, and were alleviated by LCA (Figures 4N–Q). Moreover, we evaluated myocardial cellular damage by measuring serum LDH and CK-MB levels. Obesity increased LDH levels apparently, yet LCA treatment unaffected its levels in obese mice. CK-MB is a more specific marker of myocardial cellular damage, and results showed that obesity unaffected serum CK-MB levels, yet LCA treatment reduced its levels (Figures 4R,S) in obese mice.

### LCA Supplementation Mitigates Obesity-Related Atrial Inflammatory Response

Next, we determined the transcription of pro-inflammation cytokines in the atria, including IL-1β, IL-6, IL-18, TNF-α, and MCP-1. Results demonstrated that HFD increased the transcription of IL-6 compared to STD, while LCA suppressed the transcription of most pro-inflammation cytokines, except for MCP-1, in both STD and HFD mice (Figure 4T). In addition, NFκB, a well-known downstream factor of NRF2, is a central activator of the immune response. Our results demonstrated that NFκB was activated in the atria of HFD mice, and was inhibited after LCA supplementation (Figure 4U), which is in line with our results showing LCA increased the expression and activation of NRF2 (Figures 4I–M).

### LCA Supplementation Improves Insulin Sensitivity in Obese Mice

Disordered glucose metabolism and insulin resistance are also novel risk factors for AF and usually occur in obesity (Nakamura and Sadoshima, 2020). Therefore, apart from FAs metabolism, the impacts of LCA on glycometabolism, including insulin signaling and insulin resistance, glucose and insulin tolerance, are also investigated in this study.

**FIGURE 5:**
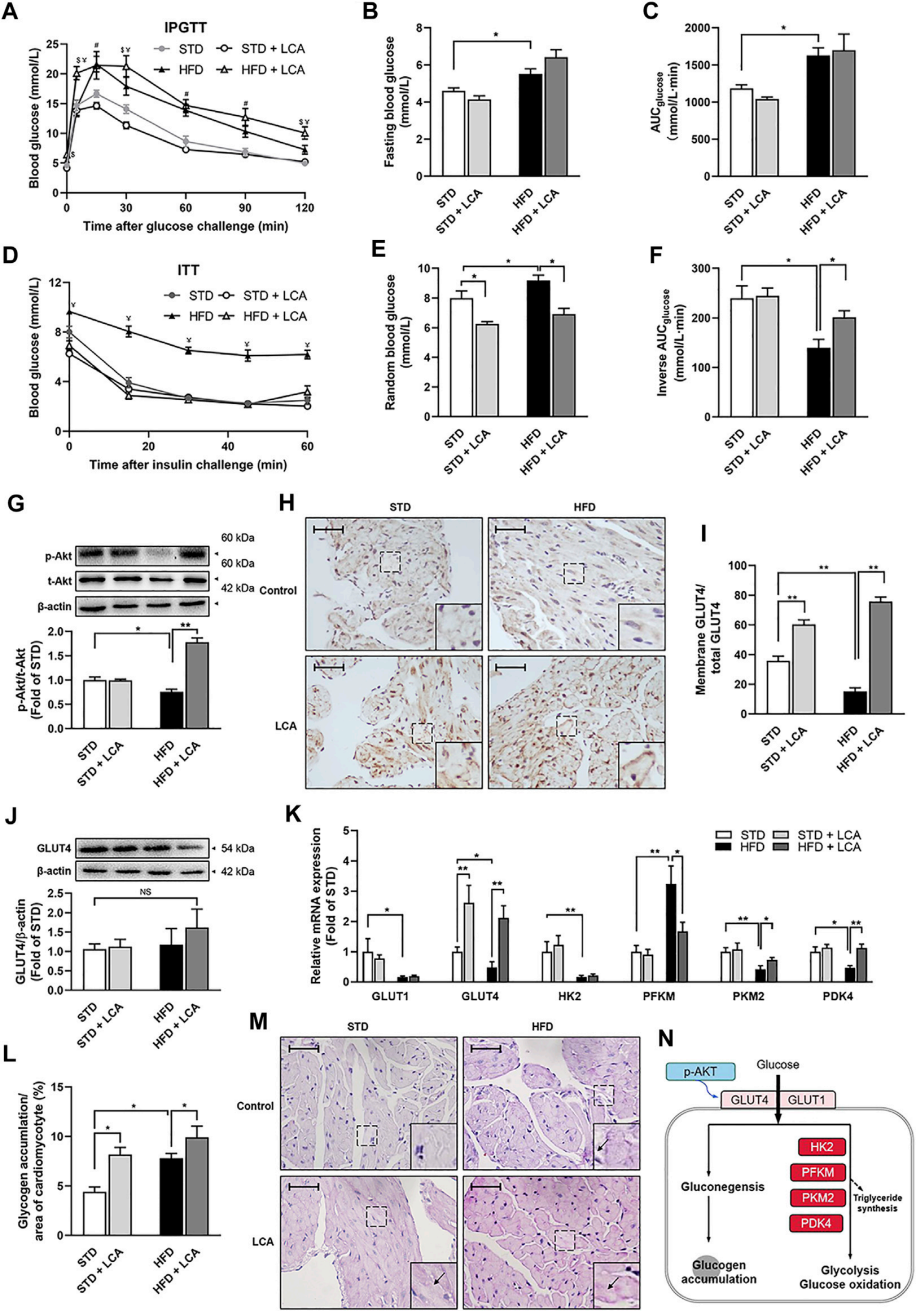
LCA restores glucose and insulin homeostasis. **(A)** Plasma glucose levels in the IPGTT. **(B)** Fast blood glucose levels among groups. **(C)** Analysis of AUC_glucose_ during IPGTT. **(D)** Plasma glucose levels in the ITT. **(E)** Random blood glucose levels among groups. **(F)** Analysis of inverse AUC_glucose_ during ITT. **(G)** Representative images and quantitative analysis of expression and phosphorylation of Akt by Western blot. **(H,I)** Representative images of membrane translocation of GLUT4 using immunohistochemistry and quantitative analyzed by ImageJ. **(J)** Representative images and quantitative analysis of the expression of GLUT4 by Western blot. **(K)** Quantitative analysis of the expression of glucose metabolism-related genes (GLUT1, GLUT4, HK2, PFKM, PKM2, and PDK4) using RT-qPCR. **(L,M)** Glycogen accumulation (Arrow) demonstrated by Periodic acid-Schiff staining. **(N)** Schematic diagram illustrating glucose metabolism. Scale bar: 50 μm *n* = 4 for *in vivo* experiments and 10 for *in vitro* experiments in each group. One-way ANOVA with Bonferroni *post-hoc* test was used to compare data among groups. Data are expressed as mean ± SEM. ^*^ *p* < 0.05, ^**^ *p* < 0.01, ^¥^ *p* < 0.05 HFD vs others, ^$^ *p* < 0.05 HFD + LCA vs others, ^#^ *p* < 0.05 STD/STD + LCA vs HFD/HFD + LCA. STD, standard diet; HFD, high-fat diet; LCA, l-carnitine; IPGTT, intraperitoneal glucose tolerance test; ITT, insulin tolerance test; AUC, area under the curve; Akt, protein kinase B; p-, phoso-; GLUT, glucose transporter; HK2, hexokinase2; PFKM, phosphofructokinase; PKM2, pyruvate kinase isozyme type M2; PDK4, pyruvate dehydrogenase kinase 4; p-, phoso-.

Both IPGTT (Figures 5A–C) and ITT (Figures 5D–F) confirmed glucose metabolic imbalance in obesity, including increased fasting blood glucose (FBG), impaired glucose tolerance, and decreased insulin sensitivity. Intriguingly, LCA had no effect on glucose tolerance and FBG, yet increased insulin tolerance in obese mice, indicating improved insulin sensitivity in peripheral tissues. Mechanistic investigation using WB analysis showed that LCA restored the serine/threonine protein kinase PKB (Akt) phosphorylation in the atria of obese mice (Figure 5G). Moreover, although LCA treatment had no effect on GLUT4 expression, its activation, as reflected by its translocation to the intracellular membrane, was promoted in obese mice (Figures 5H–J). Next, we assessed the transcription of glucose metabolism-related genes, including GLUT1, GLUT4, HK2, PFKM, PKM2, and PDK4. Most of these genes, except for PFKM, were inhibited in the atria of obese mice. LCA supplementation increased the transcription of GLUT4, PKM2, and PDK4, whereas decreased PFKM transcription in obese mice (Figure 5K). In addition, PAS staining showed aggravated glycogen accumulation in the atria of obese mice. LCA further increased glycogen accumulation in atrial cardiomyocytes (Figures 5L,M), probably due to the fact that LCA upregulated glucose uptake more than glycolysis (Figure 5N).

### Inhibition of AMPK Constrains the Cardio-Protective Effects of LCA Supplementation *in vitro*

**FIGURE 6:**
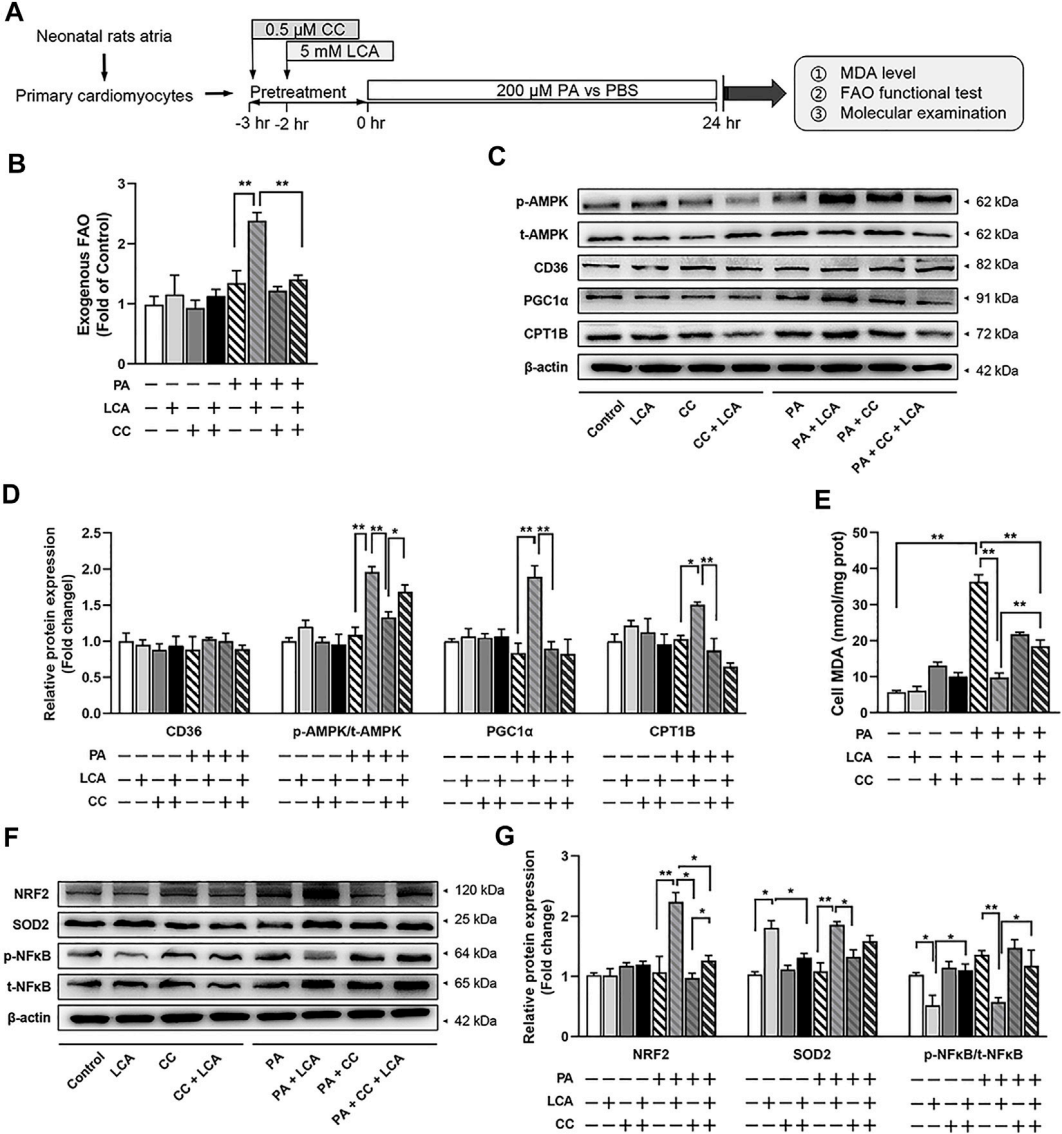
Pharmacological inhibition of AMPK via CC attenuated LCA-conferred beneficial effects in palmitate-treated primary atrial cardiomyocytes. **(A)** Schematic diagram illustrating the cell isolation, culture and treatment. **(B)** FAO rate in different treatment groups. The measure of substrate utilization after 18°C unsaturated fatty acid (Oleate, 100 μM) addition was normalized with maximal O_2_ consumption in Control cells. **(C)** Representative images and **(D)** quantitative analysis of the expression of FAO-related proteins using Western blot. **(E)** Cellular MDA concentrations among the groups. **(F)** Representative images and **(G)** quantitative analysis of the expression of oxidative stress-related proteins (NRFS and SOD2) and inflammation-related protein (NFκB) using Western blot. *n* = 3 or 4 each group. Two-way ANOVA with Bonferroni *post-hoc* test was used to compare data among groups. Data are expressed as mean ± SEM. ^*^ *p* < 0.05, ^**^ *p* < 0.01. CC, Compound C; LCA, l-carnitine; PA, palmitate; FAO, fatty acids oxidation; MDA, molondialdehyde; NRF2, nuclear erythroid 2 p45-related factor 2; AMPK, AMP-activated protein kinase; CD36, FAT; CPT1B, carnitine palmitoyltransferase-1B; p-, phoso-; PGC1α, peroxisome proliferator-activated receptor γ coactivator1α; NRF2, Nuclear factor erythroid 2-related factor 2; NFκB, the nuclear factor kappa B; SOD2, manganese superoxide dismutase, superoxide dismutase 2.

To investigate whether AMPK is crucial in LCA-conferred cardio-protection, we used PA, a saturated fatty acid, to mimic obesity *in vitro, and* CC, a pharmacological inhibitor of AMPK, to block AMPK signaling in primary atrial cardiomyocytes (Figure 6A). LCA enhanced FAO significantly in PA-treated cells, which might be ascribe to the activation AMPK and upregulation of PGC1α and CPT1B; however, inhibition of AMPK by CC reversed these effects (Figures 6C,D).

MDA examination was performed to assess the levels of oxidative stress. In line with the animal studies, LCA supplementation induced a decrement of PA-induced oxidative stress (Decreased cellular MDA) and enhanced corresponding anti-oxidative system (Restored NRF2/SOD2 signaling) in primary atrial cardiomyocytes; however, pretreatment with CC abolished LCA-conferred antioxidant effects. Similarly, pretreatment with CC also attenuated LCA-conferred anti-inflammation effects, as judged by NFκB activation (Figures 6E–G).

Altogether, these results supported that activation of AMPK signaling pathway may be the relevant molecular basis of LCA-mediated cardio-protection.

## Discussion

**FIGURE 7:**
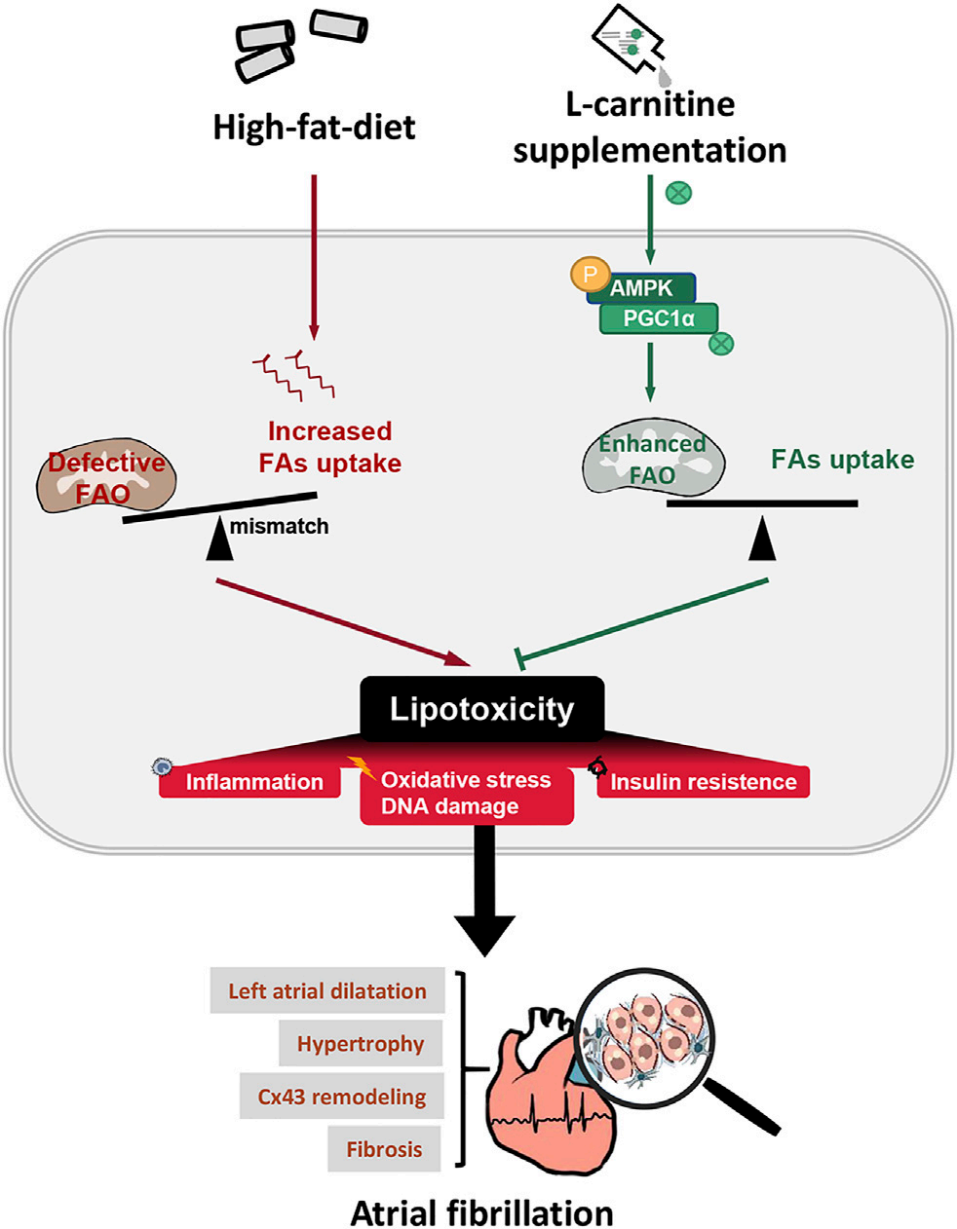
Hypothetical mechanisms of LCA-conferred cardio-protection against obesity-related AF. Possible mechanism proposed in this study: HFD-induced imbalance between FAs uptake and expenditure causes lipid accumulation and lipotoxicity, which triggers a set of chain reactions, including oxidative stress, DNA damage, inflammation and insulin resistance, and finally contributing to atrial remodeling and greater propensity for AF. Promotion of FAO via LCA combats obesity-induced AF by reducing myocardial lipotoxicity, alleviating atrial remodeling, including left atrial dilatation, cardiac hypertrophy, gap junction remodeling and interstitial fibrosis. Mechanistically, LCA supplement amended lipid metabolism through AMPK-dependent pathway. STD, standard diet; HFD, high-fat diet; LCA, l-carnitine; FAO, fatty acids oxidation; AF, atrial fibrillation; AMPK, AMP-activated protein kinase; CD36, FAT; CPT1B, carnitine palmitoyltransferase-1B; p-, phoso-; PGC1α, peroxisome proliferator-activated receptor γ coactivator1α.

Although defective FAO has long been pronounced in AF, its implication in AF is under-investigated. In this study, we established an obesity (induced by HFD)-related AF mice model, which showed increased AF vulnerability and exhibited apparent atrial structural remodeling (Figures 1, 2). Pathologically, obesity caused defective cardiac FAO and induced cardiac lipotoxicity (Figure 3) (Haffar et al., 2015), thereby evoking a set of pathological reactions, including oxidative stress, DNA damage, inflammation and insulin resistance (Figures 4, 5), which contributing to AF. Enhancing FAO *via* LCA supplementation (the cofactor of CPT1B), attenuated cardiac steatosis and lipotoxicity-induced pathological changes in the atria of obese mice, resulting in restored AF substrates and ameliorated AF susceptibility (Figure 7). Mechanistically, AMPK/PGC1α signaling was implicated in LCA-conferred beneficial effects against obesity-mediated AF (Figure 6).

The derangement of the energy substrate metabolism in the pathogenesis of AF has garnered extensive interest in the field of AF. Ample of proteomics and metabolomics studies have proved the considerable lipid metabolism remodeling in the myocardium of AF patients (Huang et al., 2011; Tu et al., 2014). Dyslipidemia is independently associated with AF incidence (Guan et al., 2020), and lipid metabolism related proteins serve as a potential AF biomarker (such as LDL, VLDL, HDL, and FABP3) (Golaszewska et al., 2019). Enzymes involved in FAO (such as CD36, CPT1B and VLCAD), as well as their regulators (such as PPAR-α and PGC1α), are demonstrated to inhibited in chronic AF (Liu et al., 2016). These evidence supported the impaired FAs uptake and defective FAO, along with increased atrial lipid deposition of AF (Lenski et al., 2015), suggesting disordered lipid metabolism is closely related to the occurrence and development of AF (Opacic et al., 2016). Consequently, from a broader metabolic perspective, redressing the disbalance of lipid metabolism should be considered as a novel a candidate strategy for AF. Supportively, AMPK, an effective lipid metabolism accelerator, and its downstream effectors, PPAR-α/PGC-1α signals, both have been proposed as alternative metabolic modulations to combat AF (Harada et al., 2015; Bai et al., 2019; Ostropolets et al., 2021). Genetic deletion of liver kinase B1, an activator of AMPK, can develop spontaneous AF in mice (Ozcan et al., 2015). Besides, in agreement with inactivated AMPK signaling in atria in long-standing AF (Harada et al., 2015), restoring FAO targeting AMPK, PPAR-α or PGC1α (Metformin, AICAR, Fenofibrate and β_3_AR) (Yu et al., 2011; Liu et al., 2016; Bai et al., 2019; Deshmukh et al., 2021; Ostropolets et al., 2021) have been confirmed to suppress AF inducibility. However, AMPK, a key regulator of multi-pathways and multi-targets, also gets involved in inflammation mitigation, Ca^2+^-handling and cell contraction, mitochondrial biogenesis, cell growth and proliferation, and so on, thus exerting cardiovascular protection with a combination of multiple mechanisms (Garcia and Shaw, 2017). Therefore, the cardio-protective effect of AMPK-mediated pathways cannot be differentiated from FAO promotion. It is worthy to evaluated the direct effects of accelerated FAO on AF, especially in circumstances of obesity, in which cardiac FAO is defective considerably compared to other well-known ‘culprits’ of the AF. As expected, we proved that boosting FAO *via* LCA, a natural and biologically active micronutrient enhancing physiological FAO through CPT1B (Marcovina et al., 2013) and activation of AMPK signaling pathway (Figures 3, 6), can significantly reduce obesity-mediated AF propensity and the corresponding atrial remodeling (Figures 1, 2), thus better supports the efficiency of FAO promotion in the AF therapeutic approach.

Whereas, in opposite to our theory, FAO suppression *via* Ranolazine has been demonstrated to attenuate AF occurrence in 1-week ACh-CaCl_2_-exposed rats (Zou et al., 2016), probably by supporting the transient supply/demand mismatch during the stabilization of AF (Heijman and Dobrev, 2015). This controversial result can be further explained by the nonspecific confounding influence of Ranolazine, since it directly blocks the late sodium channel to terminate arrhythmia, and attenuates adverse myocardial alterations including hypertrophy and fibrosis (De Angelis et al., 2016). More notably, prolonged inhibition of FAO would presumably increase cardiac preference for carbohydrate sources, which represents a driving force for atrial electrical remodeling as well as followed irreversible structural remodeling after a long period (Kolwicz and Tian, 2011); and in addition induces atrial lipid accumulation, which promotes lipotoxicity-provoked AF (Young et al., 2002; Lundsgaard et al., 2020).

Over-accumulation of lipid in cardiomyocyte cytosol occurs when FAs supply fails to match the needs created by FAs expenditure, thus is commonly observed in obesity (Haffar et al., 2015) as well as AF (Lenski et al., 2015). Notably, cardiac steatosis in atria exerts detrimental impacts on heart (termed “lipotoxicity”), thus taking a leading role in AF, especially in obesity-related AF (Haffar et al., 2015; Opacic et al., 2016). For instance, excess myocardial FAs can convert into potentially “lipotoxic” metabolites, such as diacylglycerol and ceramides (Serra et al., 2013), which directly affect excitation-contraction coupling and ion channel/pump integrity, and later contribute to irreversible structure alterations (Harada et al., 2017). Besides, lipid-derived excessive oxidants generation and impaired antioxidant capacity (inactivated NRF2-cascade) lead to redox imbalance and trigger inflammatory response (Li et al., 2019), which are in line with progressively deterioration of myocardial structure and function (Goldberg et al., 2012), including LA enlargement, and myocardial hypertrophy, connexin-43 remodeling, interstitial fibrosis in the atria (Harada et al., 2017). What’s more, cardiac lipotoxicity causes insulin dysregulation and glycometabolism impairment (Nakamura and Sadoshima, 2020), which are speculated to provide a metabolic arrhythmogenic substrate for AF (Maria et al., 2018). Cardiac lipotoxicity induced by HFD was further delineated in this study, and ameliorated after FAO promotion which redressed the disbalance of lipid metabolism and normalized the cardiac lipid content, thus explained the correlation between defective FAO and AF/obesity (Figures 3–5).

Disordered glucose metabolism and insulin homeostasis are also active metabolic subjects in the study of AF. Prior researchers have established strong correlation between inadequate glycemic control and AF episodes (Dublin et al., 2010). Within cardiomyocytes, suppressed atrial glucose oxidation and increased glycogen synthesis occur in AF and promote marked glycogen accumulation (Heijman and Dobrev, 2015). Besides, insulin resistance is considered as a novel independent risk factor for AF, which engenders both atrial structural remodeling and abnormal intracellular calcium homeostasis (Chan et al., 2019; Wu et al., 2014). What’s more, insulin signaling loss, noted as impaired glucose transport (alterations in the expression and trafficking of GLUT4), has been proposed to be an early pathogenic factor of AF pathogenesis (Maria et al., 2018). Intra-myocardial toxic metabolites of FAs metabolism and triacylglycerol, such as diacylglycerol, ceramides and acylcarnitines, are responsible for severe insulin resistance by interrupting insulin signal cascade at different levels and multiple steps of glucose metabolism (Belfort et al., 2005; Yazici and Sezer, 2017). In consistent to ameliorated lipid accumulation and lipotoxicity, enhanced FAO *via* LCA promoted insulin-stimulated glucose uptake, thus restored random blood glucose in obesity (Figure 5). Some other experimental studies have given another explanation for the improved carbohydrate metabolism, that LCA supplementation can reduce the ratio of acetyl-CoA to free CoA in the mitochondria, thereby stimulating the activity of pyruvate dehydrogenase (PDH) (Calvani et al., 2000). However, our results implied the activated Akt and upregulated subsequent glucose utilization in the atria may be the reason why LCA supplementation can recover the glucose metabolism and insulin homeostasis, and thus expanding the potential role of FAO promotion *via* LCA in AF suppression.

Although present observations have unveiled the practical meaning of promoted FAO *via* LCA in obesity-dependent AF, and indicating the leading role of FAO in AF pathogenesis, this study failed to directly assess the efficiency of FAs uptake or FAO *in vivo*, and whether the LCA-conferred anti-AF effects is mediated by AMPK also needs confirmation *in vivo*. Further investigative work is warranted to address the limitation.

## Conclusion

In conclusion, we present for the first time that FAO promotion *via* LCA could redress lipid metabolism imbalance and reduce cardiac lipotoxicity through AMPK activation, thereby ameliorating obesity-mediated AF and atrial structural remodeling. Enhancing FAO may optimize the therapeutic strategy for AF, especially in obesity.

## Data Availability

The original contributions presented in the study are included in the article/Supplementary Material, further inquiries can be directed to the corresponding authors.

## Abbreviations

AF, atrial fibrillation; AMPK, AMP-activated protein kinase; CPT1B, carnitine palmitoyltransferase-1B; CC, compound C; FAO, fatty acids oxidation; FAs, fatty acids; HFD, high-fat diet; LCA, L-carnitine; NRF2, nuclear factor erythroid 2-related factor2; PGC1α, peroxisome proliferator-activated receptor γ coactivator1α; STD, standard diet.
