# Supplementary material for: Enhancing Fatty Acids Oxidation via L-Carnitine Attenuates Obesity-Related Atrial Fibrillation and Structural Remodeling by Activating AMPK Signaling and Alleviating Cardiac Lipotoxicity
Source: Front Pharmacol. 2021 Nov 26;12:771940. doi: 10.3389/fphar.2021.771940 (PMC8662783; doi:10.3389/fphar.2021.771940)
Supplement: Supplementary file 1 [file DataSheet2.pdf]

Table S1. Primers used for RT-qPCR amplification

Gene	Forward Primer (5' to 3')	Reverse Primer (5' to 3')
Hypertrophy-related gene		
β-MHC	CGAGTCCCAGGTCAACAAG	AGGCTCTTTCTGCTGGACC
Fibrosis-related gene		
α-SMA	GCAAACAGGAATACGACGAAGC	GCTTTGGGCAGGAATGATTTG
Collagen I	GAGCGGAGAGTACTGGATCG	TACTCGAACGGGAATCCATC
Collagen III	ACGTAAGCACTGGTGGACAG	GGAGGGCCATAGCTGAACTG
Smad3	CCTTCTGGTGCTCCATCTCC	ACCTCTCCCAATGTGTCGC
Smad7	CCTCGGAAGTCAAGAGGCTG	CAGCCTGCAGTTGGTTTGAG
TGF-β	CTGTCCAAACTAAGGCTCGC	AGACAGCCACTCAGGCGTAT
Inflammation-related gene		
MCP-1	CAGGTCCCTGTCATGCTTCT	TCTGGACCCATTCCTTCTTG
IL-6	CTTCCATCCAGTTGCCTTCTTG	AATTAAGCCTCCGACTTGTGAAG
IL-1β	TGGTGTGTGACGTTCCCATT	TCGTTGCTTGGTTCTCCTTG
IL-18	GCCGACTTCACTGTACAACCG	GAGGGTCACAGCCAGTCCTC
TNF-α	AGGGCTGTGGGACCTAAATGT	ATGGGATGAGTATGGGGCAGC
Metabolism-related gene		
CD36	CGGAACTGTGGGCTCATTG	GCATGAGAATGCCTCCAAACA
CPT1B	GTGCAAGCAGCCCGTCAG	TTGCGGCGATACATGATCAT
FABP-pm	CGAGCAGGGCATCAATGTCT	CCGTACAGGCCCATCTTCTT
FABP3	ACTCGGTGTGGGCTTTGC	ATGATGGTAGTAGGCTTGGTCATG
GLUT1	GTGTATCCTGTTGCCCTTC	GCTTCTTCAGCACACTCTT
GLUT4	ATGGCTGTCGCTGGTTTCTC	ACCCATGCCGACAATGAAGT
HK2	GGAACCCAGCTGTTTGACCA	CAGGGGAACGAGAAGGTGAAA
PDK4	AGCTGGTGAAGAGTACAAAT	TCTGGTTGAAGAGCTGGTATATC
PFKM	GCCATCGCCGTGTTGAC	GCCCTGACGGCAGCATT
PGC1α	CGGAAATCATATCCAACCAG	TGAGGACCGCTAGCAAGTTTG
PKM2	TGACTCTGCCCCCATCA	GCAGGCCCAATGGTACAAAT
β-actin	ACCTCTATGCCAACACAGTG	GGACTCATCGTACTCCTGCT

β-MHC, β–cardiac myosin heavy chain; α-SMA, α-smooth muscle actin; Smad, drosophila mothers against decapentaplegic protein; TGF-β, transform growth factor-β; MCP-1, monocyte chemoattractant protein-1; IL, interleukin; TNF-α, tumor necrosis factor-α; CD36, FAT; CPT1B, carnitine palmitoylransferase-1B; FABP-pm, plasma membrane fatty acid-binding protein; FABP3, fatty acid binding protein 3; GLUT, glucose transporter; HK2, hexokinase2; PDK4, pyruvate dehydrogenase kinase 4; PFKM, phosphofructokinase; PGC1α, peroxisome proliferator-activated receptor γ coactivator1α; PKM2, pyruvate kinase isozyme type M2.

Table S2. Surface ECG and Echocardiographic parameters of LV among STD, STD + LCA, HFD and HFD + LCA group

Parameter	STD (n = 10)	STD + LCA (n = 10)	HFD (n = 10)	HFD + LCA (n = 10)
Echocardiography				
Aorta root (mm)	1.68 ± 0.03	1.64 ± 0.07	1.70 ± 0.05	1.73 ± 0.07
IVS_d (mm)	0.85 ± 0.03	0.91 ± 0.05	0.97 ± 0.05*	1.06 ± 0.04†
IVS_s (mm)	1.18 ± 0.06	1.37 ± 0.06*	1.45 ± 0.05*	1.43 ± 0.05
$LVID_d$ (mm)	3.64 ± 0.06	3.63 ± 0.13	3.58 ± 0.10	3.42 ± 0.08
$LVID_s$ (mm)	2.45 ± 0.09	2.48 ± 0.72	2.31 ± 0.14	2.34 ± 0.15
$LVPW_d$ (mm)	0.70 ± 0.03	0.77 ± 0.04	0.87 ± 0.05*	0.75 ± 0.05†
$LVPW_s$ (mm)	1.20 ± 0.08	1.03 ± 0.06†	1.28 ± 0.07	1.20 ± 0.09
LV mass (mg)	96.05 ± 4.46	107.98 ± 6.87	121.29 ± 7.02*	108.89 ± 5.60†
$_{\text{Corrected}}$ LV mass (mg)	76.84 ± 3.57	86.38 ± 5.50	97.12 ± 5.60*	87.11 ± 4.48†
EF (%)	61.84 ± 2.43	60.00 ± 5.74	64.65 ± 2.83	60.34 ± 4.38
FS (%)	27.56 ± 4.85	32.92 ± 3.23	35.20 ± 2.20	32.20 ± 3.23
Surface ECG				
PR interval (ms)	51.68 ± 2.78	44.76 ± 1.67	47.85 ± 3.42	44.25 ± 3.00
QRS duration (ms)	25.83 ± 0.96	20.05 ± 0.87*	25.00 ± 1.37	19.32 ± 1.90†
QT_cB interval (ms)	110.68 ± 2.77	89.02 ± 4.27*	115.82 ± 3.19	93.38 ± 9.45†

n = 10 per group. One-way ANOVA was used to compare STD, STD + LCA, HFD, and HFD + LCA group data. The data were expressed as mean ± SEM. $^*P < .05$ vs STD, $^†P < .05$ vs HFD. AF, atrial fibrillation; AO, aortic root diameter; ECG, electrocardiogram; EF, ejection fraction; FS, fraction shortening; HFD, high-fat diet; IVS_d, end-diastolic inter-ventricular septum thickness; IVSs, end-systolic inter-ventricular septum thickness; LCA, L-carnitine; LV, left ventricle; $LVID_d$, end-diastolic LV internal dimension; $LVID_s$, end-systolic LV internal dimension; $LVPW_d$, end-diastolic LV posterior wall thickness; $LVPW_s$, end-systolic LV posterior wall thickness; STD, standard diet.
