# Supplementary material for: Enhancing Fatty Acids Oxidation via L-Carnitine Attenuates Obesity-Related Atrial Fibrillation and Structural Remodeling by Activating AMPK Signaling and Alleviating Cardiac Lipotoxicity
Source: Front Pharmacol. 2021 Nov 26;12:771940. doi: 10.3389/fphar.2021.771940 (PMC8662783; doi:10.3389/fphar.2021.771940)
Supplement: Supplementary file 2 [file DataSheet3.pdf]

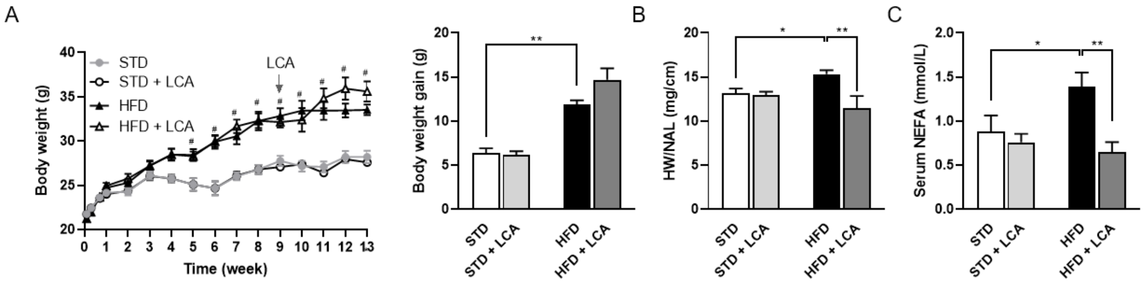

Figure S1. LCA supplementation attenuates heart weight gain and dyslipidemia in obesity. **(A)** Comparisons of BW alteration, BW gain, **(B)** HW and **(C)** serum NEFA among groups. Thirteen weeks HFD establishes an obesity-induced AF mouse model. HFD increases BW, HW and serum NEFA as compared to STD. LCA (150 mg/kg·BW/d) supplementation decreased HW and serum NEFA, but had no obvious effect on BW in obese mice.n = 10 per group. One-way ANOVA with Bonferroni *post-hoc test* was used to compare data among STD, STD + LCA, HFD, and HFD + LCA groups. Data are expressed as mean ± SEM. $^{*}P < 0.05$, $^{**}P < 0.001$, $^{\#}P < 0.05$ STD/STD + LCA vs HFD/HFD + LCA. STD, standard diet; HFD, high-fat diet; LCA, L-carnitine; FAO, fatty acids oxidation; BW, body weight; HW, heart weight; NAL, naso-anal length; NEFA, non-esterified fatty acids.

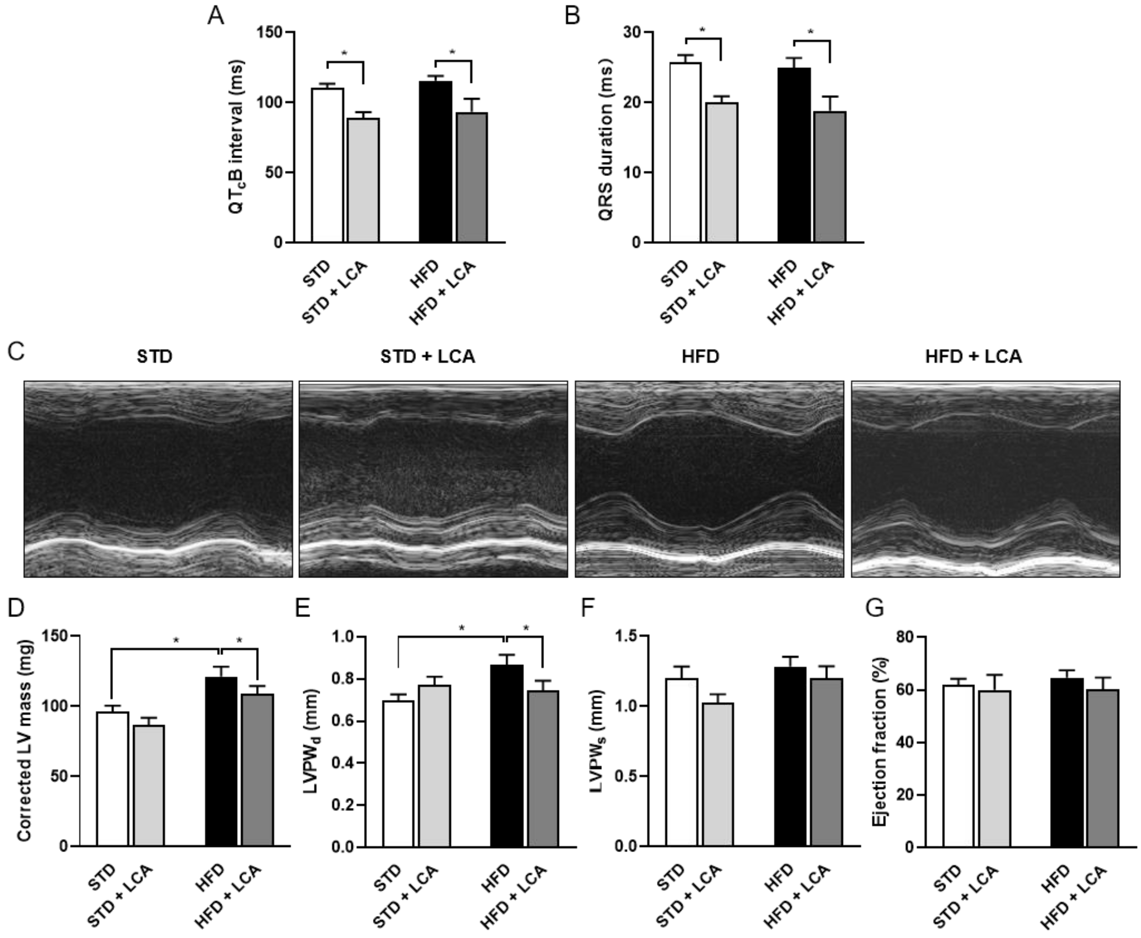

Figure S2. LCA supplementation improves electrical changes and attenuates ventricular hypertrophy in obesity. **(A)** Analysis of QT$_c$B interval and **(B)** QRS duration. **(C)** Representative echocardiographic images of LV among the groups. **(D-G)** Measurements of $_{\text{corrected}}$ LV mass, LVPW$_d$, LVPW$_s$ and EF detected by 2D-guided M-mode imaging. n = 10 per group. One-way ANOVA with Bonferroni *post-hoc* test was used to compare data among STD, STD + LCA, HFD, and HFD + LCA groups. Data are expressed as mean ± SEM. $^{*}P < 0.05$, $^{\#}P < 0.05$ STD/STD + LCA vs HFD/HFD + LCA. STD, standard diet; HFD, high-fat diet; LCA, L-carnitine; FAO, fatty acids oxidation; LV, left ventricle; IVS, inter-ventricular septum; LVPW$_d$, end-diastolic LV posterior wall thickness; LVPW$_s$, end-systolic LV posterior wall thickness; EF, ejection fraction.
