# Supplementary material for: Enhancing Fatty Acids Oxidation via L-Carnitine Attenuates Obesity-Related Atrial Fibrillation and Structural Remodeling by Activating AMPK Signaling and Alleviating Cardiac Lipotoxicity
Source: Front Pharmacol. 2021 Nov 26;12:771940. doi: 10.3389/fphar.2021.771940 (PMC8662783; doi:10.3389/fphar.2021.771940)
Supplement: Supplementary file 3 [file DataSheet1.PDF]

Example of original western blot (shown by 'B&P Example of original western blot for three repeats' online)

Figure 1. Example of original western blot for three repeats

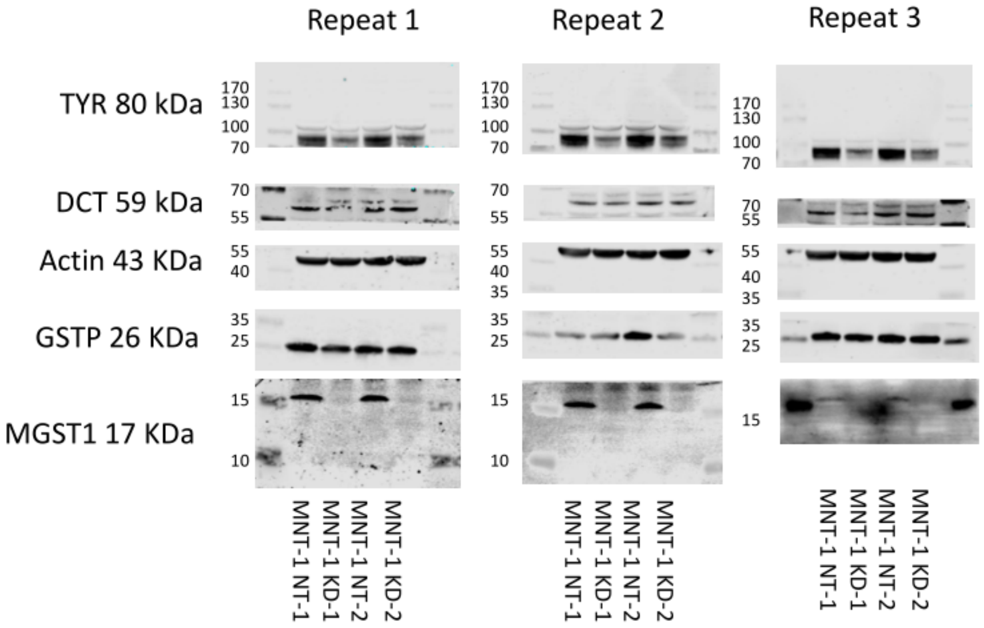

If the whole membrane was cut to blot for different antibodies, you are required to load all pieces of whole blot and the molecular marker must be shown in each blot. Figure 1 shows the whole blot after cutting membrane at molecular weight 70 kDa, 55 kDa, 35 kDa and 15~25 kDa for TYR (80 kDa), DCT (59 kDa), actin (43 kDa), GSTP (26 kDa) and MGST1 (17 kDa).

Material S1. Original images of western blot for three repeats of mice sample.

Representative images of atrial remodeling involved proteins (TGFβ, αSMA, Collagen I, CollagenIII, Connexin-43 and Connexin-40), metabolism-related molecules (CD36, AMPK, PGC1α, CPT1B and Akt) and NRF2-cascade (NRF2, SOD2 and NFκB) by Western blot in standard diet (STD), STD + L-carnitine (STD + LCA), high-fat-diet (HFD), HFD + L-carnitine (HFD + LCA) group (Unedited gel images for Figures 2-5).

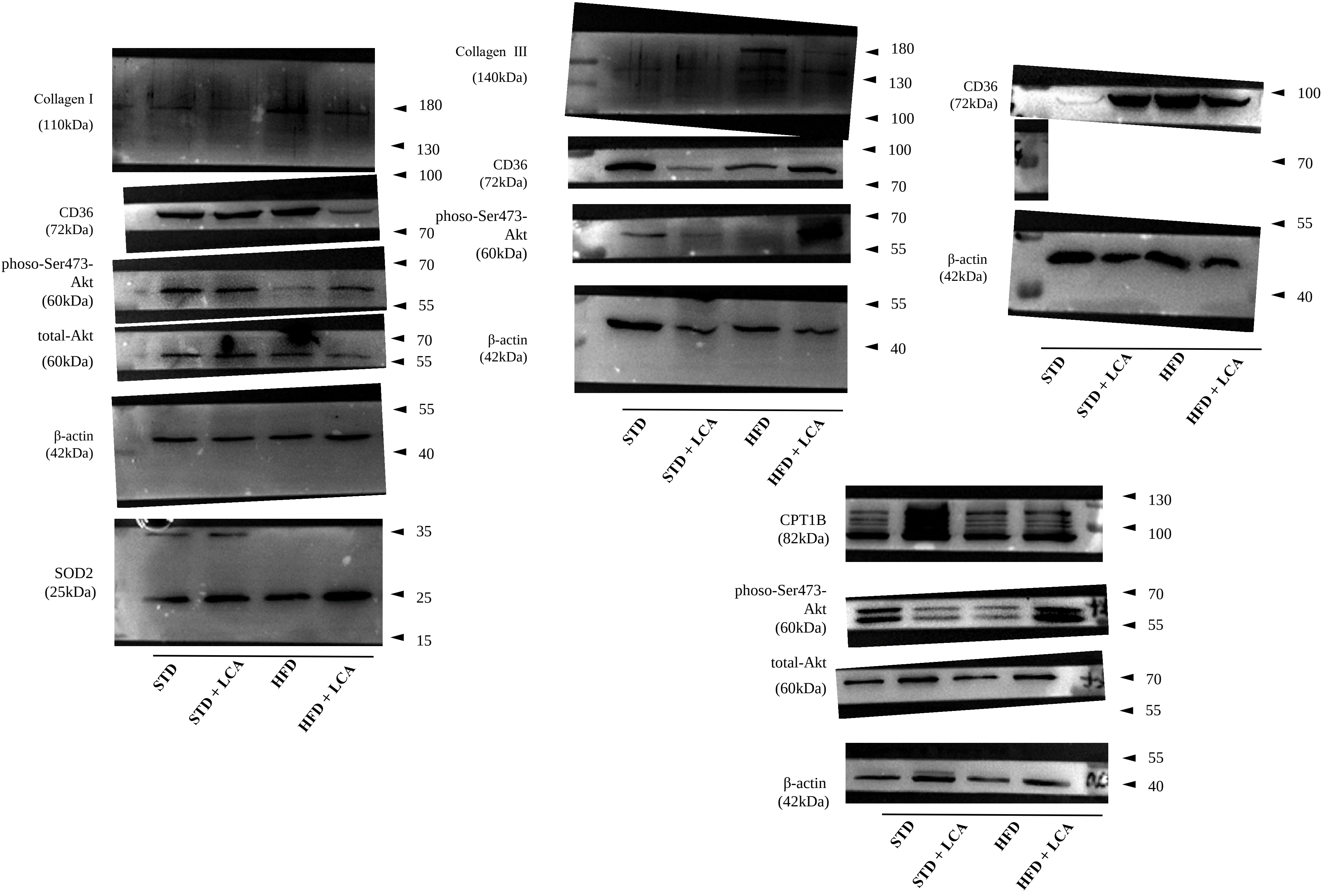

Collagen I
(110kDa)
180
130
100
CD36
(72kDa)
70
phoso-Ser473-
Akt
(60kDa)
70
55
total-Akt
(60kDa)
70
55
β-actin
(42kDa)
55
40
SOD2
(25kDa)
35
25
15
STD
STD + LCA
HFD
HFD + LCA
Collagen III
(140kDa)
180
130
100
CD36
(72kDa)
100
70
phoso-Ser473-
Akt
(60kDa)
70
55
β-actin
(42kDa)
55
40
STD
STD + LCA
HFD
HFD + LCA
CD36
(72kDa)
100
70
β-actin
(42kDa)
55
40
STD
STD + LCA
HFD
HFD + LCA
CPT1B
(82kDa)
130
100
phoso-Ser473-
Akt
(60kDa)
70
55
total-Akt
(60kDa)
70
55
β-actin
(42kDa)
55
40
STD
STD + LCA
HFD
HFD + LCA

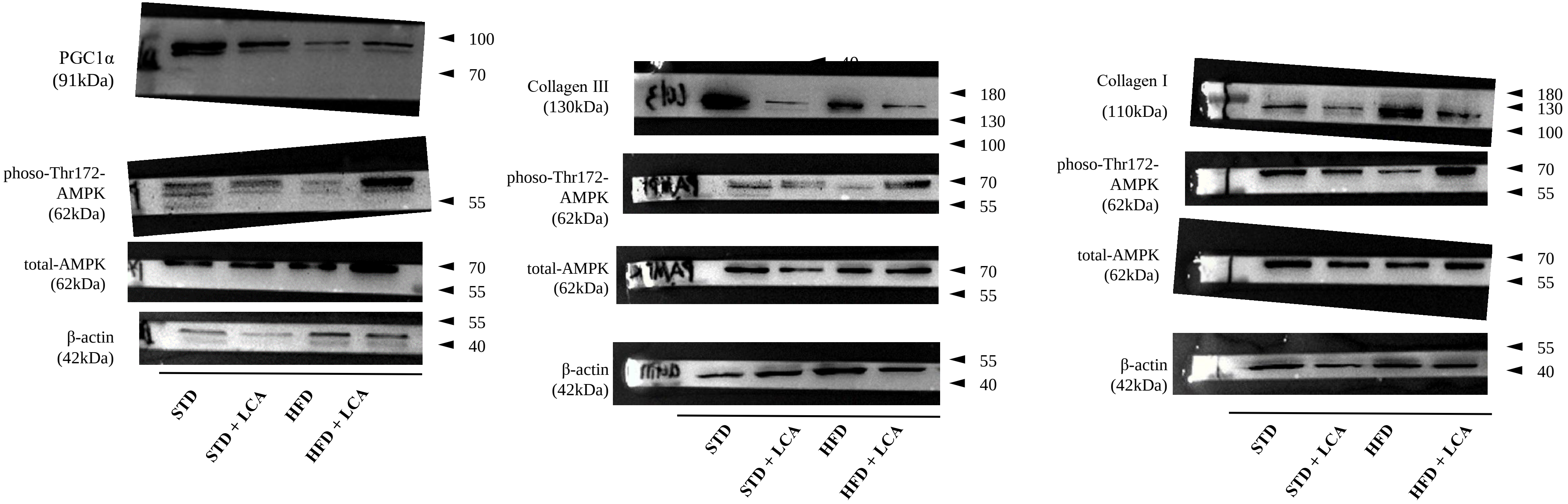
PGC1α
(91kDa)
100
70
phoso-Thr172-
AMPK
(62kDa)
55
total-AMPK
(62kDa)
70
55
β-actin
(42kDa)
55
40
STD
STD + LCA
HFD
HFD + LCA
Collagen III
(130kDa)
180
130
100
phoso-Thr172-
AMPK
(62kDa)
70
55
total-AMPK
(62kDa)
70
55
β-actin
(42kDa)
55
40
STD
STD + LCA
HFD
HFD + LCA
Collagen I
(110kDa)
180
130
100
phoso-Thr172-
AMPK
(62kDa)
70
55
total-AMPK
(62kDa)
70
55
β-actin
(42kDa)
55
40
STD
STD + LCA
HFD
HFD + LCA

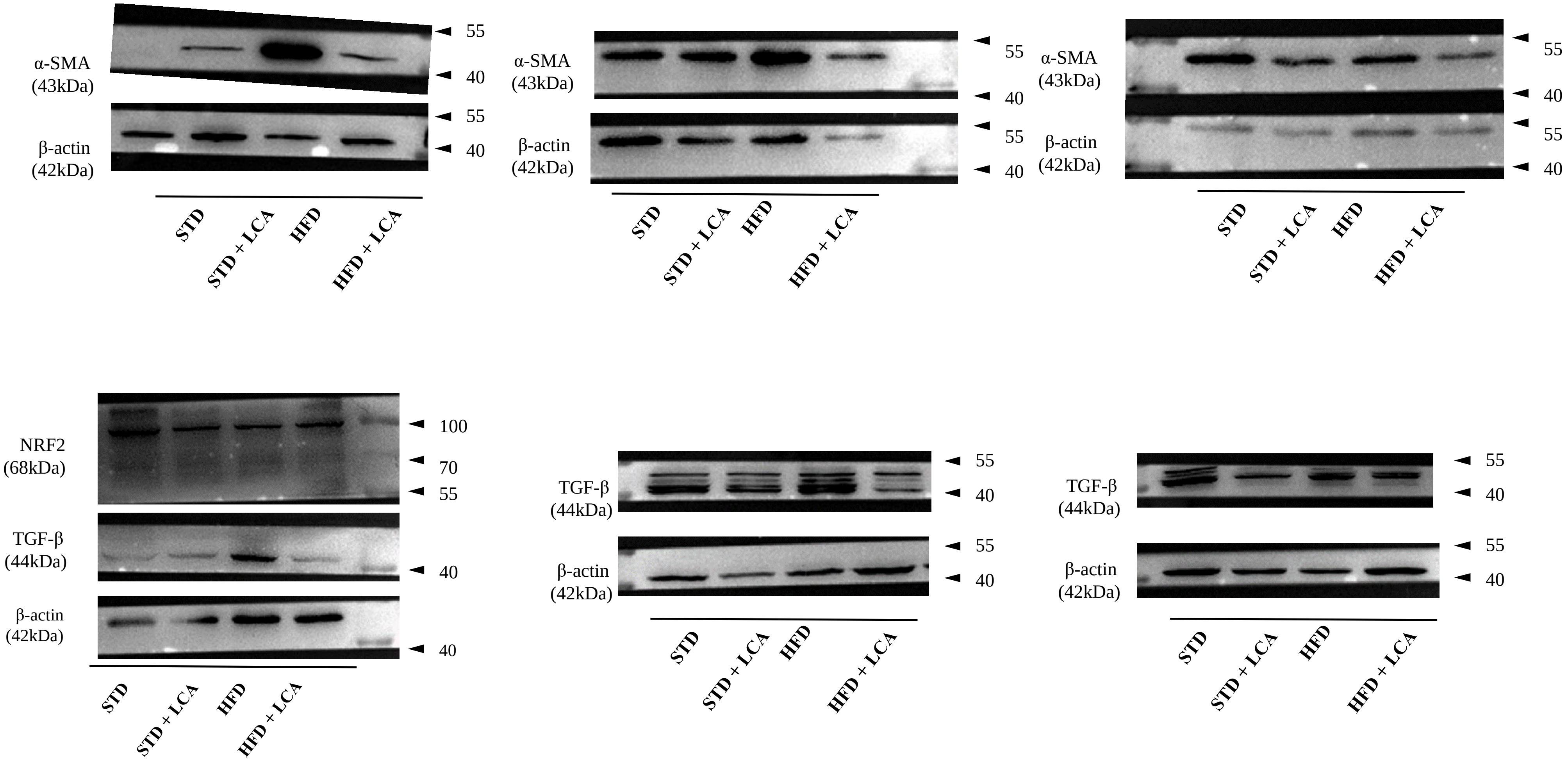
α-SMA
(43kDa)
β-actin
(42kDa)
55
40
STD
STD + LCA
HFD
HFD + LCA
NRF2
(68kDa)
TGF-β
(44kDa)
100
70

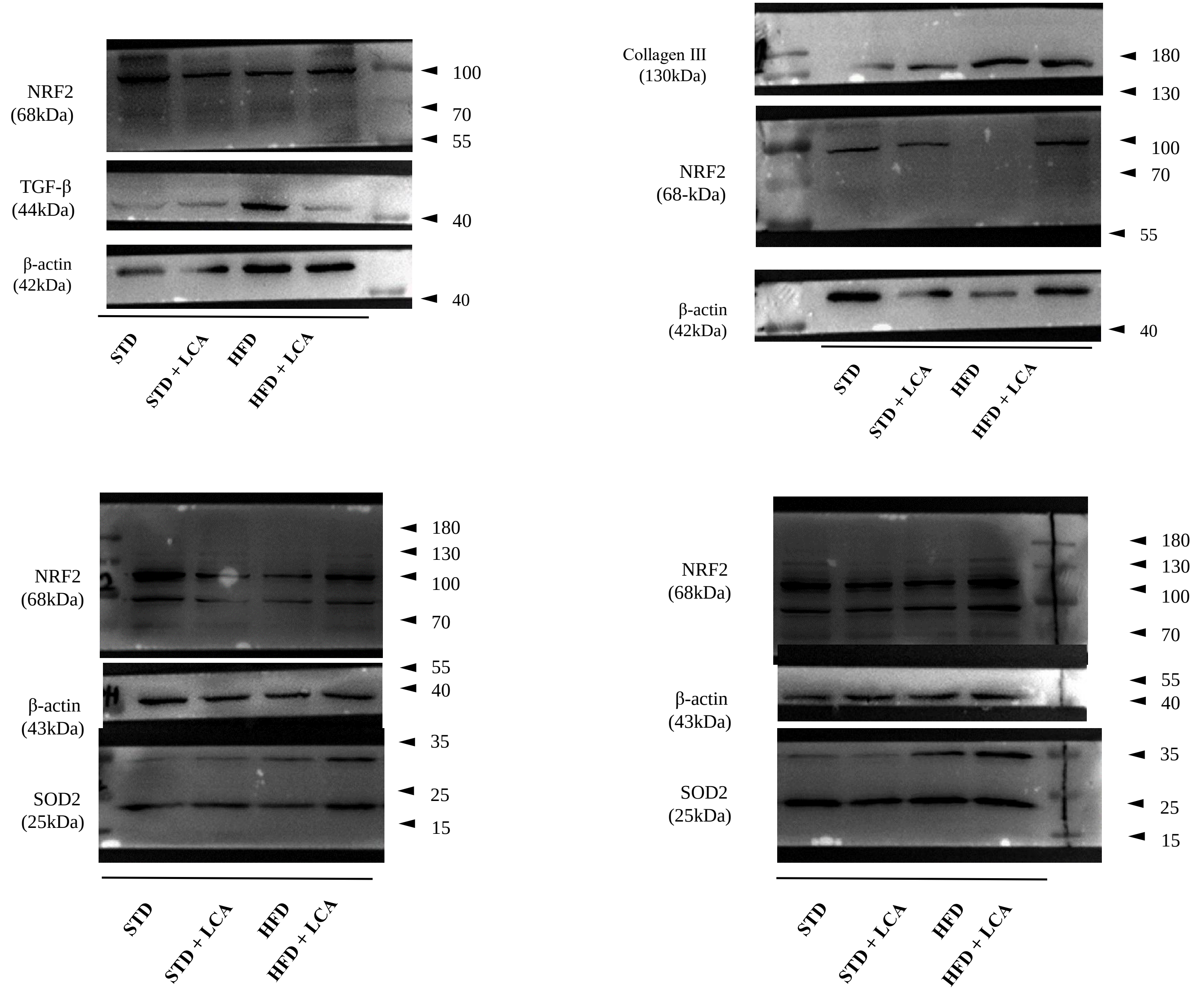
NRF2 (68kDa)
TGF-β (44kDa)
β-actin (42kDa)
100
70
55
40
40
STD
STD + LCA
HFD
HFD + LCA
Collagen III (130kDa)
NRF2 (68-kDa)
β-actin (42kDa)
180
130
100
70
55
40
STD
STD + LCA
HFD
HFD + LCA
NRF2 (68kDa)
β-actin (43kDa)
SOD2 (25kDa)
180
130
100
70
55
40
35
25
15
STD
STD + LCA
HFD
HFD + LCA
NRF2 (68kDa)
β-actin (43kDa)
SOD2 (25kDa)
180
130
100
70
55
40
35
25
15
STD
STD + LCA
HFD
HFD + LCA

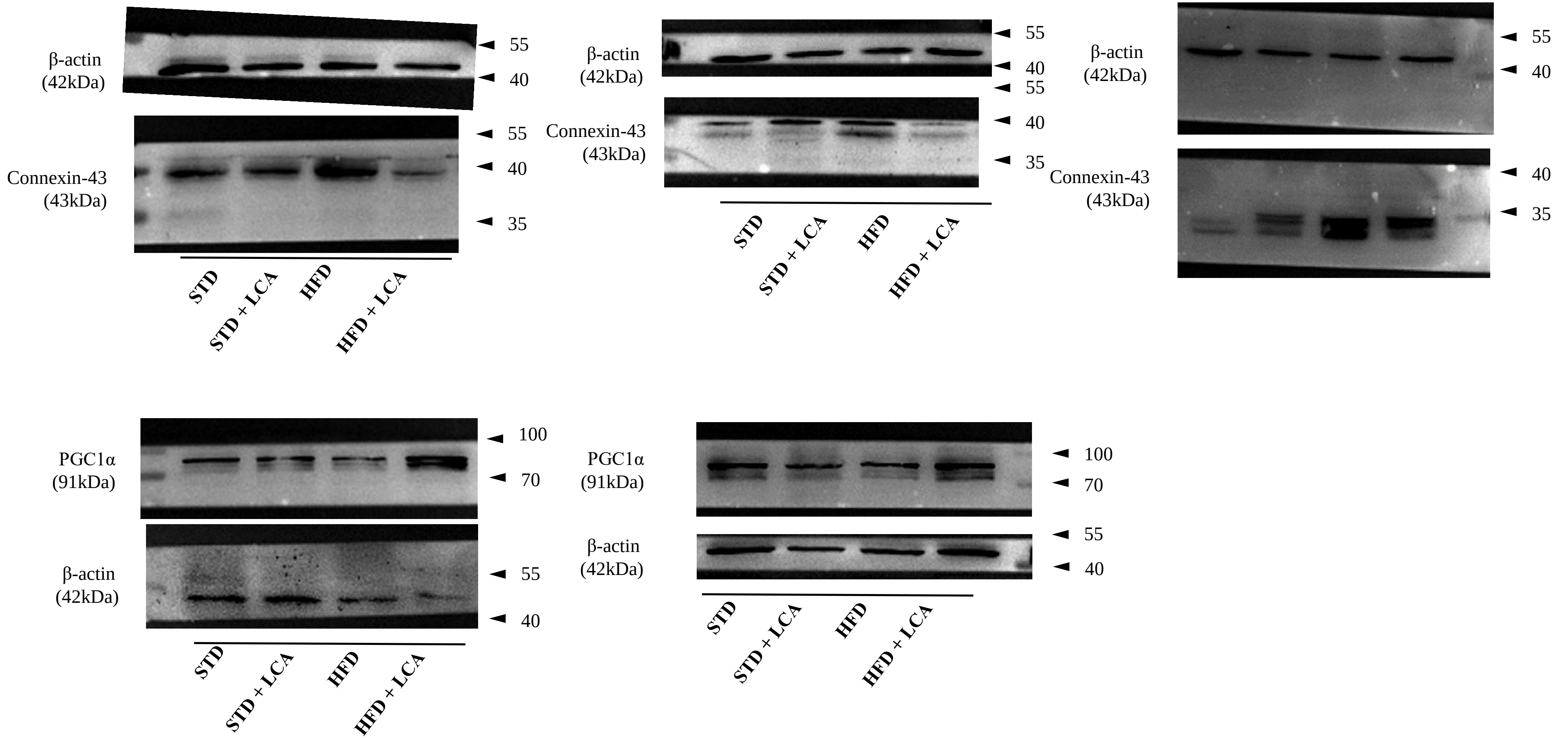
β-actin
(42kDa)
Connexin-43
(43kDa)
55
40
35
STD
STD + LCA
HFD
HFD + LCA
PGC1α
(91kDa)
100
70

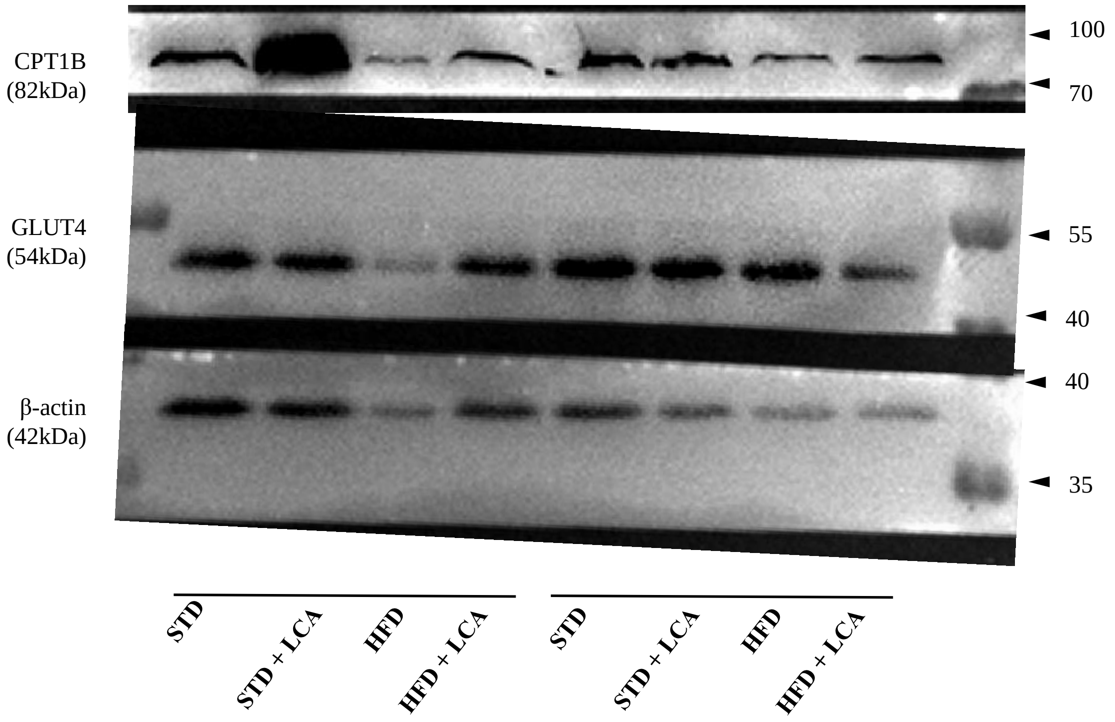
CPT1B
(82kDa)
GLUT4
(54kDa)
β-actin
(42kDa)
100
70
55
40
40
35
STD
STD + LCA
HFD
HFD + LCA
STD
STD + LCA
HFD
HFD + LCA

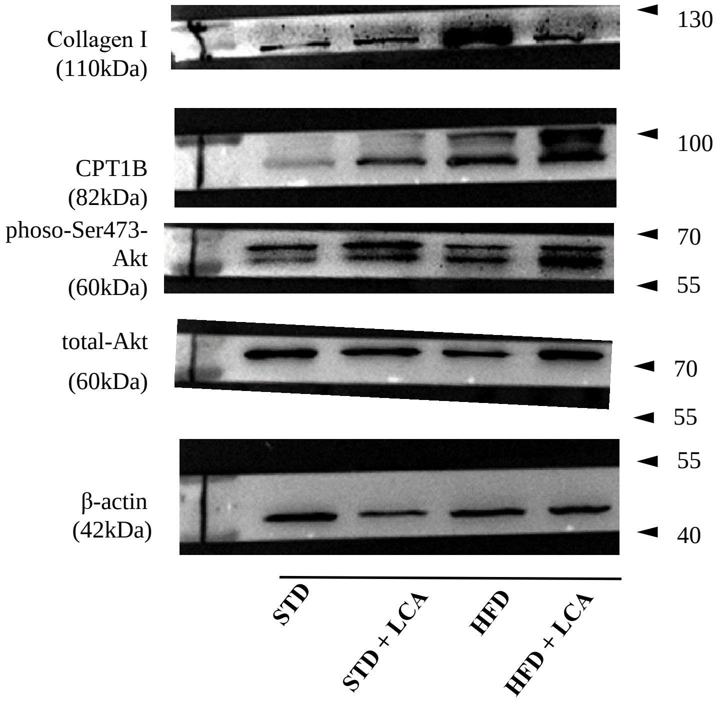
Collagen I
(110kDa)
CPT1B
(82kDa)
phoso-Ser473-
Akt
(60kDa)
total-Akt
(60kDa)
β-actin
(42kDa)
130
100
70
55
70
55
55
40
STD
STD + LCA
HFD
HFD + LCA

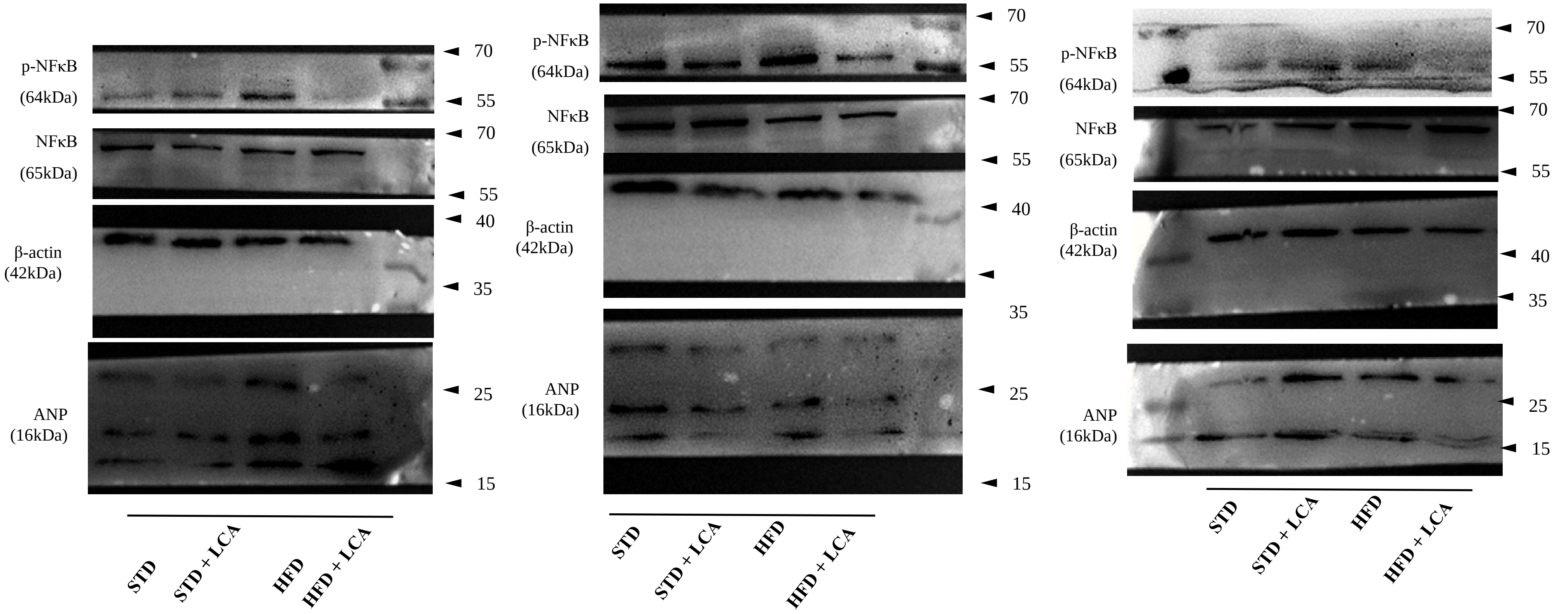
p-NFκB
(64kDa)
NFκB
(65kDa)
β-actin
(42kDa)
ANP
(16kDa)
70
55
40
35
25
15
STD
STD + LCA
HFD
HFD + LCA

Material S3. Original images of western blot for three repeats of cell sample (Primary cardiomyocytes).

Representative images of lipid ocidation-related molecules (CD36, AMPK, PGC1α and CPT1B) and NRF2-cascade (NRF2, SOD2 and NFκB) by Western blot in control, control + L-carnitine (LCA), control + compound C (CC), control + L-carnitine + compound C (LCA + CC), palmitic acid (PA), palmitic acid + L-carnitine (PA + LCA), palmitic acid + compound C (PA + CC), palmitic acid + L-carnitine + compound C (PA + LCA + CC) group (Unedited gel images for Figure 6).

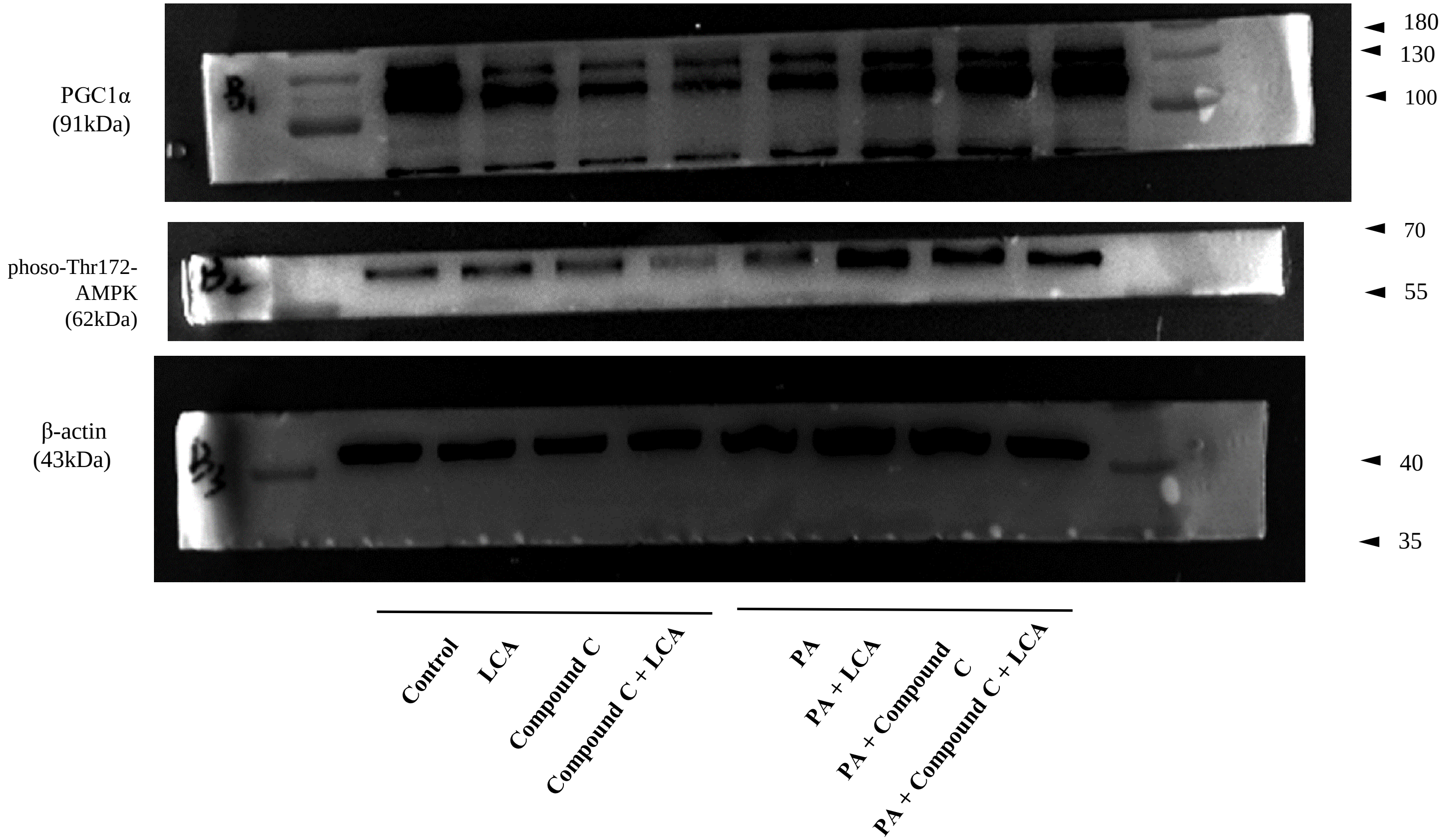

PGC1α
(91kDa)
phoso-Thr172-
AMPK
(62kDa)
β-actin
(43kDa)
180
130
100
70
55
40
35
Control
LCA
Compound C
Compound C + LCA
PA
PA + LCA
PA + Compound
C
PA + Compound C + LCA

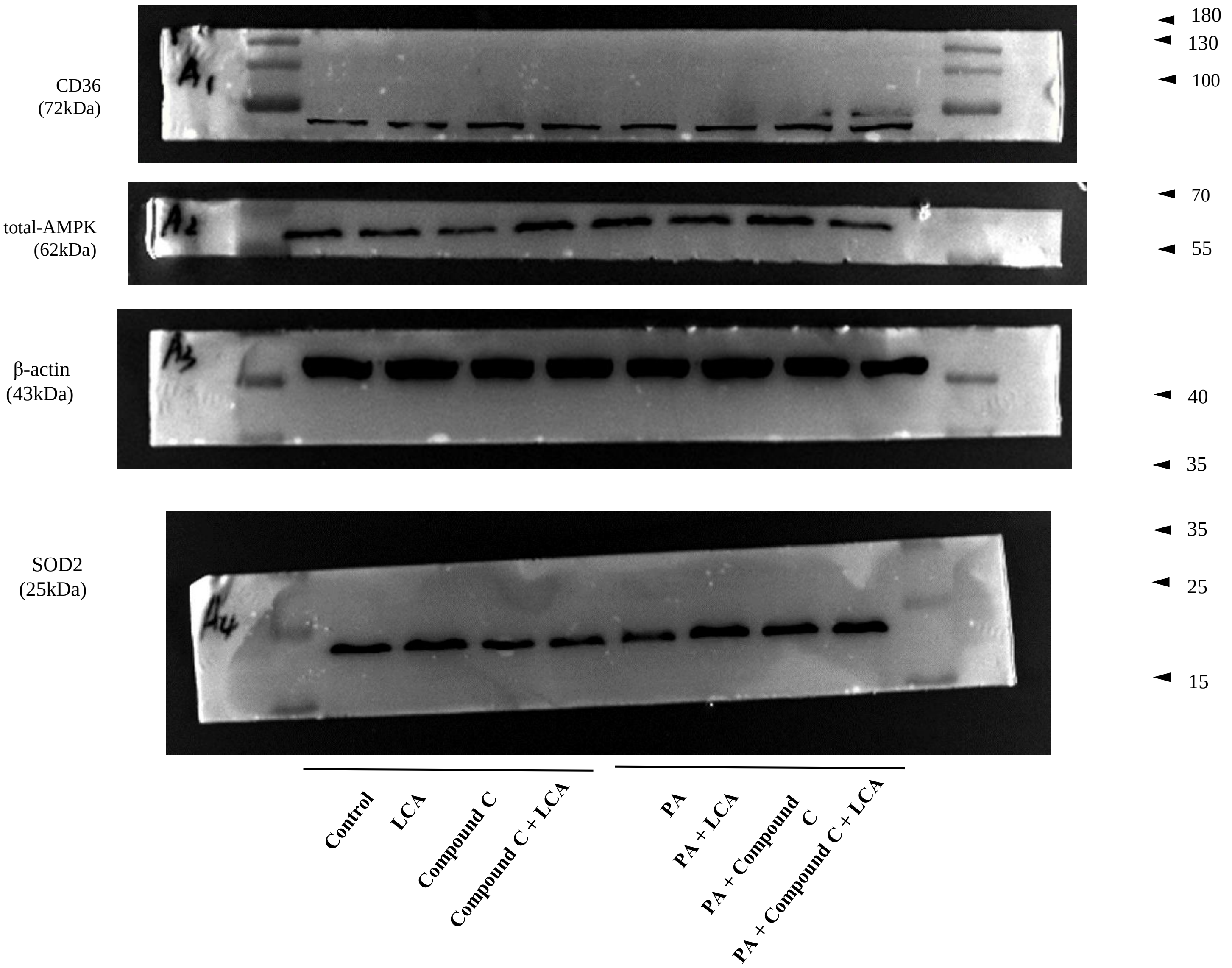

CD36
(72kDa)
total-AMPK
(62kDa)
β-actin
(43kDa)
SOD2
(25kDa)
180
130
100
70
55
40
35
35
25
15
Control
LCA
Compound C
Compound C + LCA
PA
PA + LCA
PA + Compound C
PA + Compound C + LCA

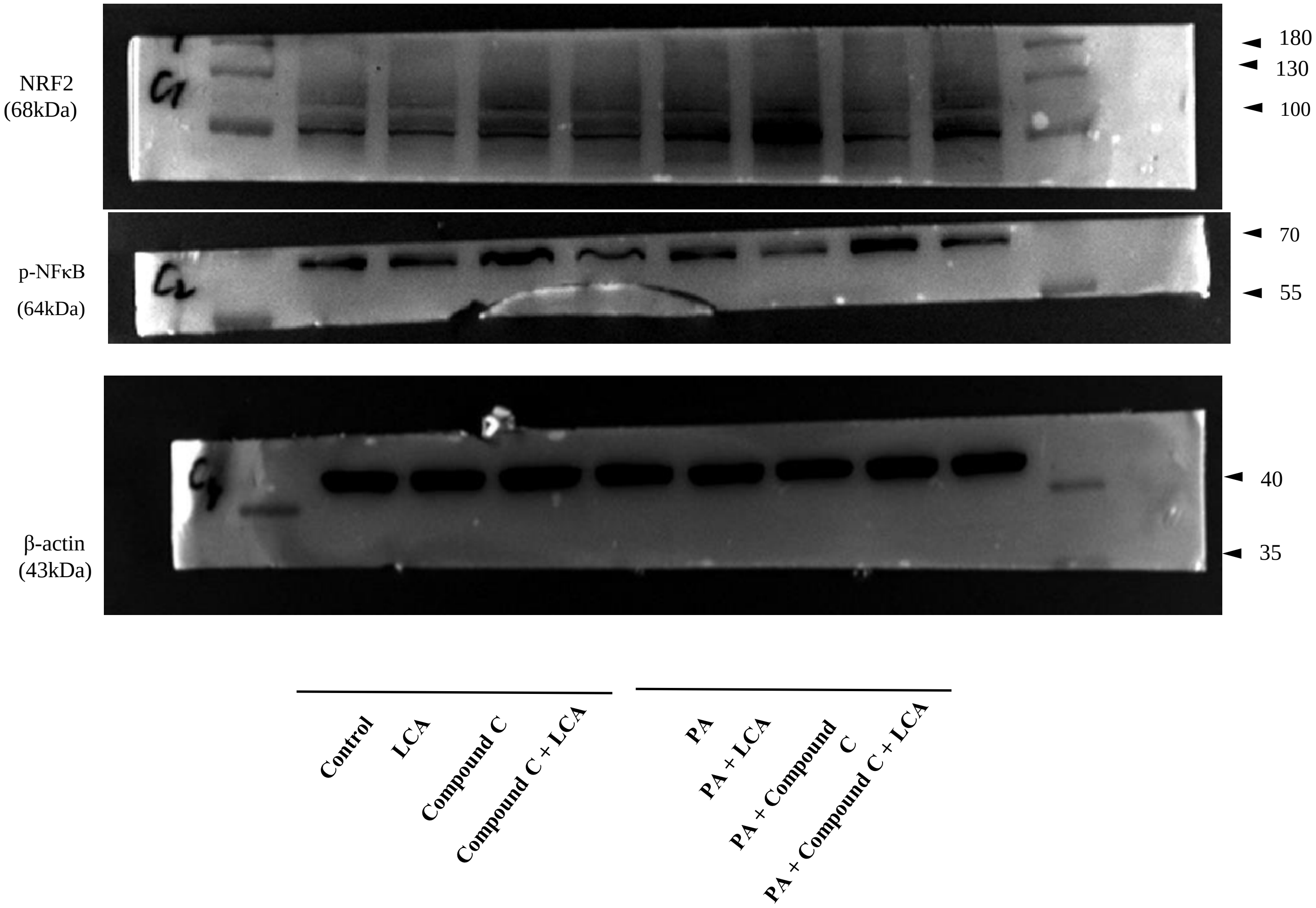

NRF2
(68kDa)
p-NFκB
(64kDa)
β-actin
(43kDa)
180
130
100
70
55
40
35
Control
LCA
Compound C
Compound C + LCA
PA
PA + LCA
PA + Compound C
PA + Compound C + LCA

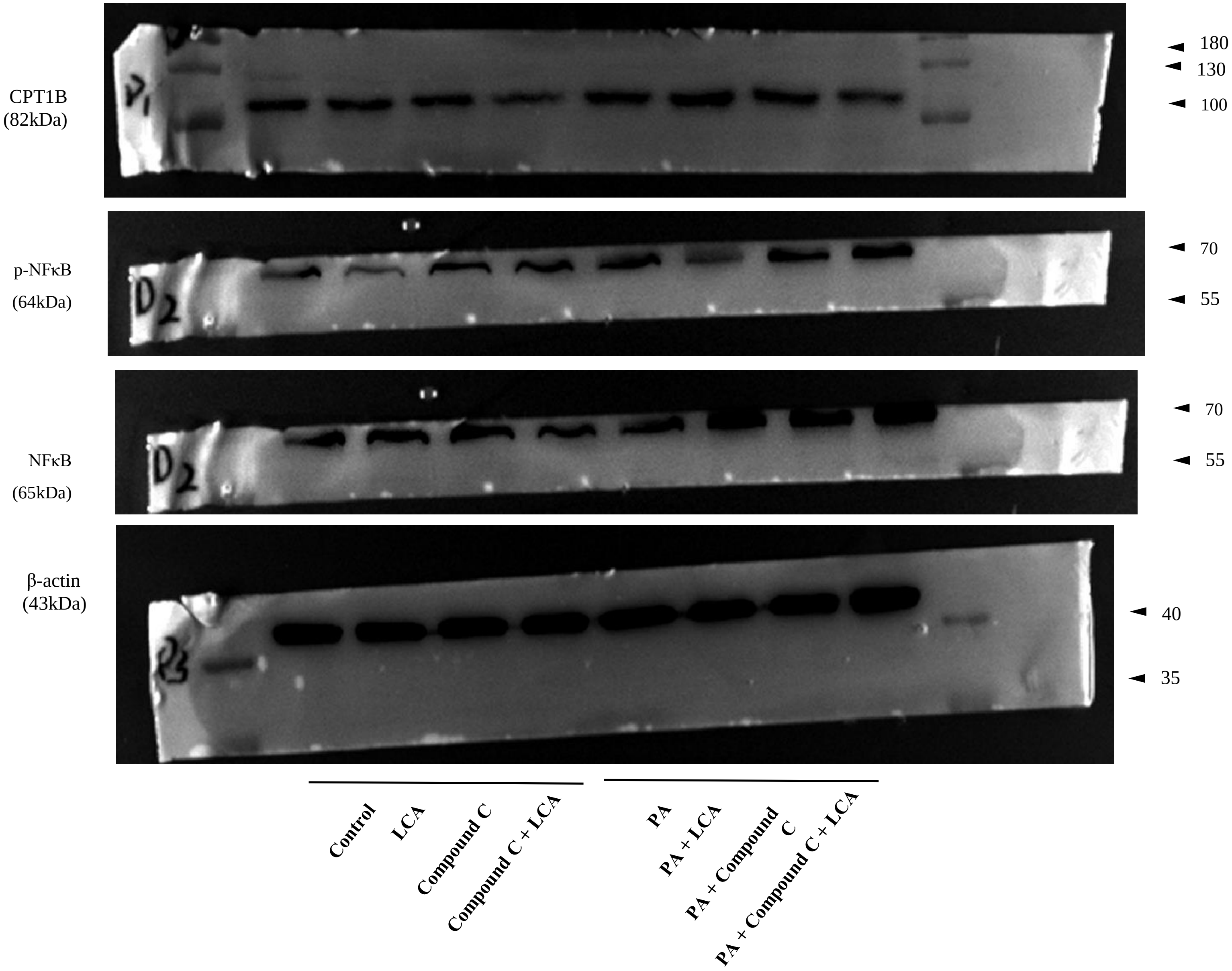
CPT1B
(82kDa)
p-NFκB
(64kDa)
NFκB
(65kDa)
β-actin
(43kDa)
180
130
100
70
55
70
55
40
35
Control
LCA
Compound C
Compound C + LCA
PA
PA + LCA
PA + Compound C
PA + Compound C + LCA

Material S2. Original images of western blot for three repeats of cell sample (HL-1 cell lines).

Representative images of lipid ocidation-related molecules (CD36, AMPK, PGC1α and CPT1B) and NRF2-cascade (NRF2, SOD2 and NFκB) by Western blot in control, control + L-carnitine (LCA), control + compound C (CC), control + L-carnitine + compound C (LCA + CC), palmitic acid (PA), palmitic acid + L-carnitine (PA + LCA), palmitic acid + compound C (PA + CC), palmitic acid + L-carnitine + compound C (PA + LCA + CC) group (Unedited gel images for Figure 6).

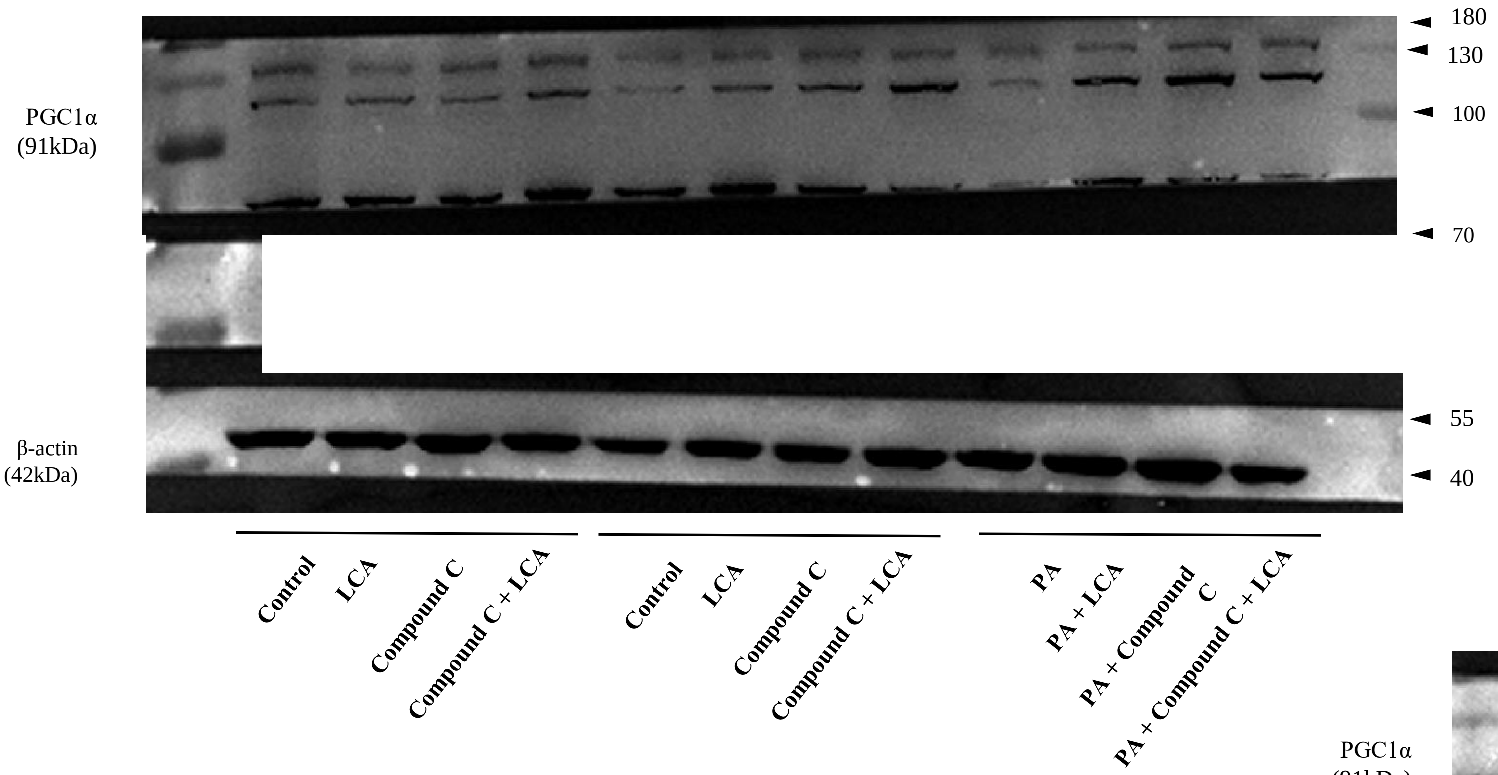
PGC1α
(91kDa)
β-actin
(42kDa)
180
130
100
70
55
40
Control
LCA
Compound C
Compound C + LCA
Control
LCA
Compound C
Compound C + LCA
PA
PA + LCA
PA + Compound C
PA + Compound C + LCA

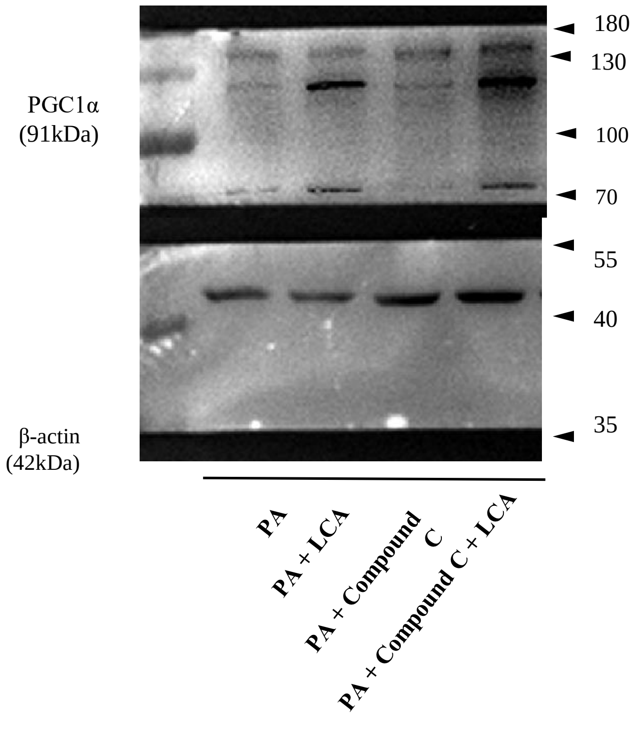
PGC1α
(91kDa)
β-actin
(42kDa)
180
130
100
70
55
40
35
PA
PA + LCA
PA + Compound C
PA + Compound C + LCA

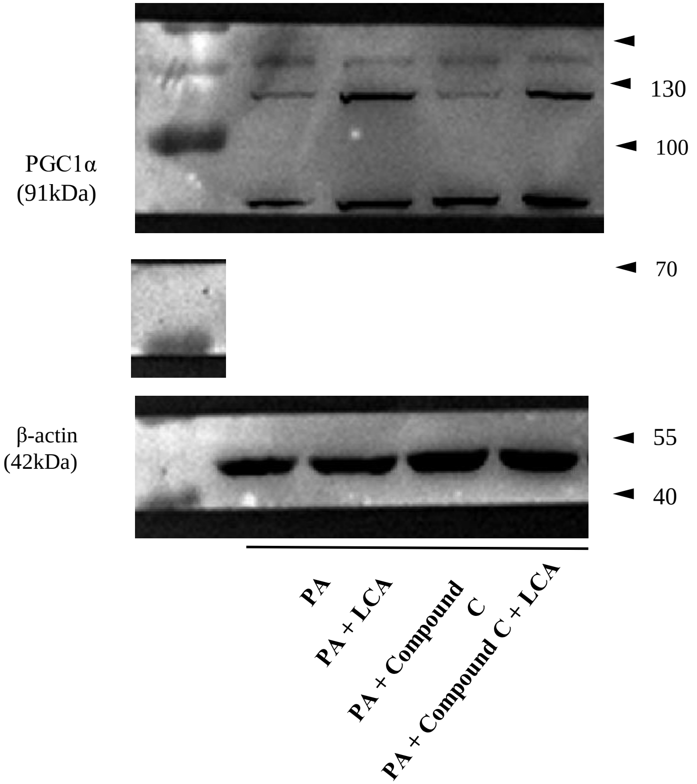
180
PGC1α
(91kDa)
130
100
70
β-actin
(42kDa)
55
40
PA
PA + LCA
PA + Compound C
PA + Compound C + LCA

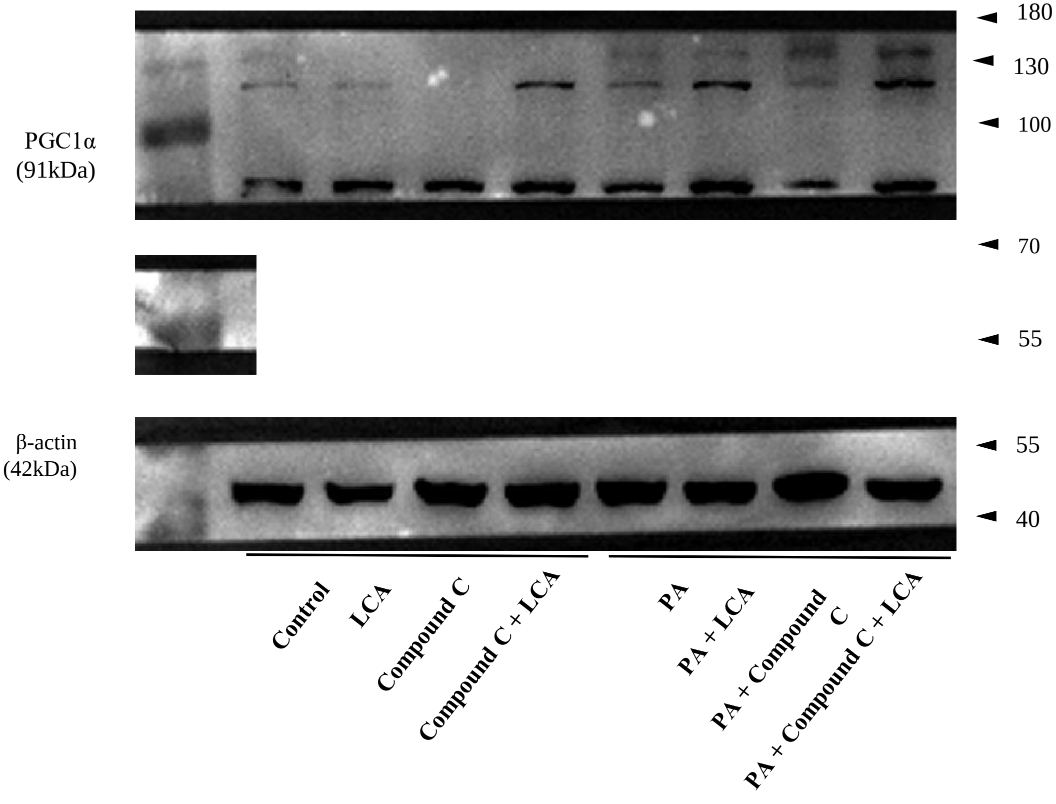
180
130
100
PGC1α
(91kDa)
70
55
β-actin
(42kDa)
55
40
Control
LCA
Compound C
Compound C + LCA
PA
PA + LCA
PA + Compound C
PA + Compound C + LCA

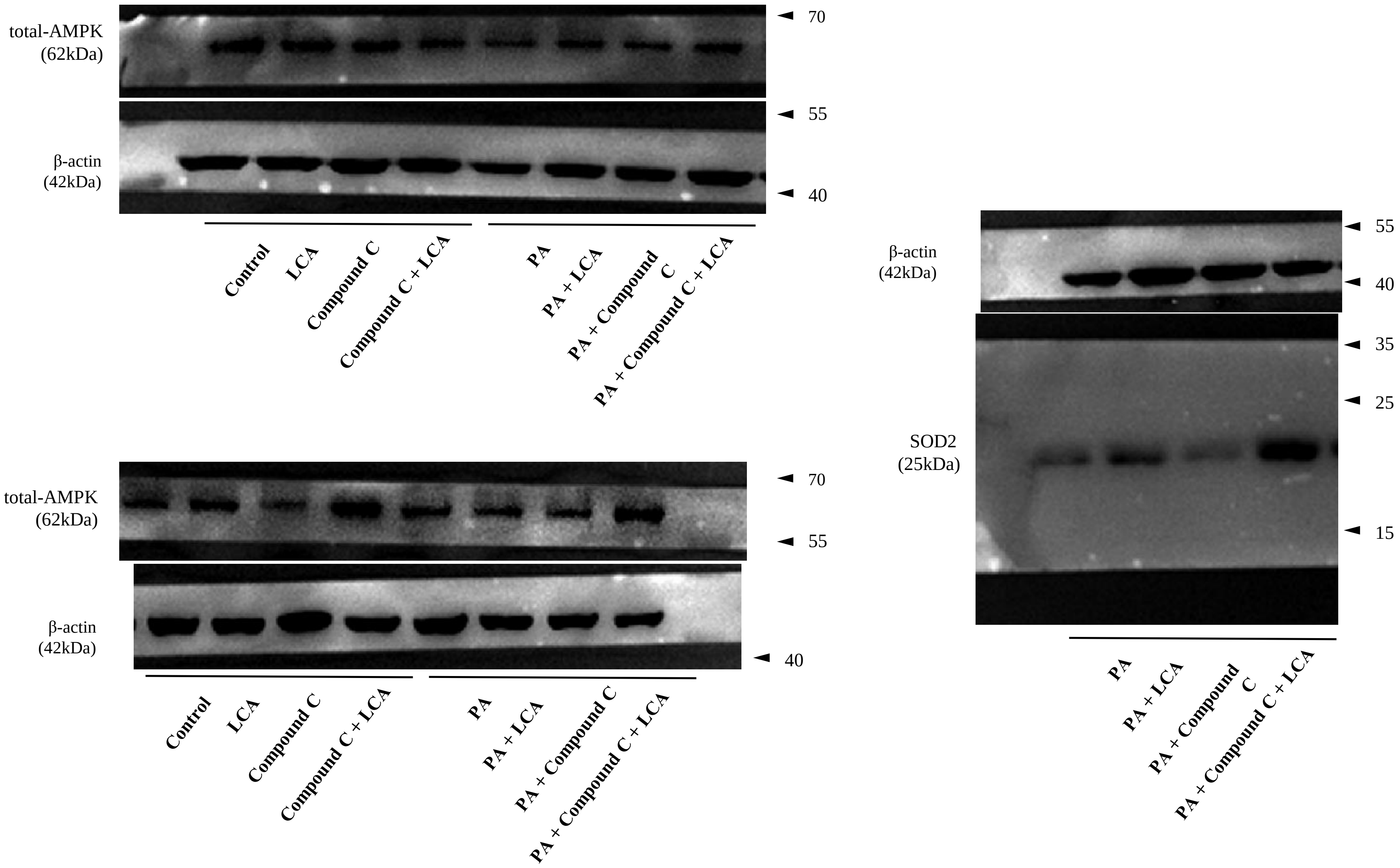
total-AMPK
(62kDa)
β-actin
(42kDa)
70
55
40
Control
LCA
Compound C
Compound C + LCA
PA
PA + LCA
PA + Compound C
PA + Compound C + LCA
total-AMPK
(62kDa)
β-actin
(42kDa)
70
55
40
Control
LCA
Compound C
Compound C + LCA
PA
PA + LCA
PA + Compound C
PA + Compound C + LCA
β-actin
(42kDa)
SOD2
(25kDa)
55
40
35
25
15
PA
PA + LCA
PA + Compound C
PA + Compound C + LCA

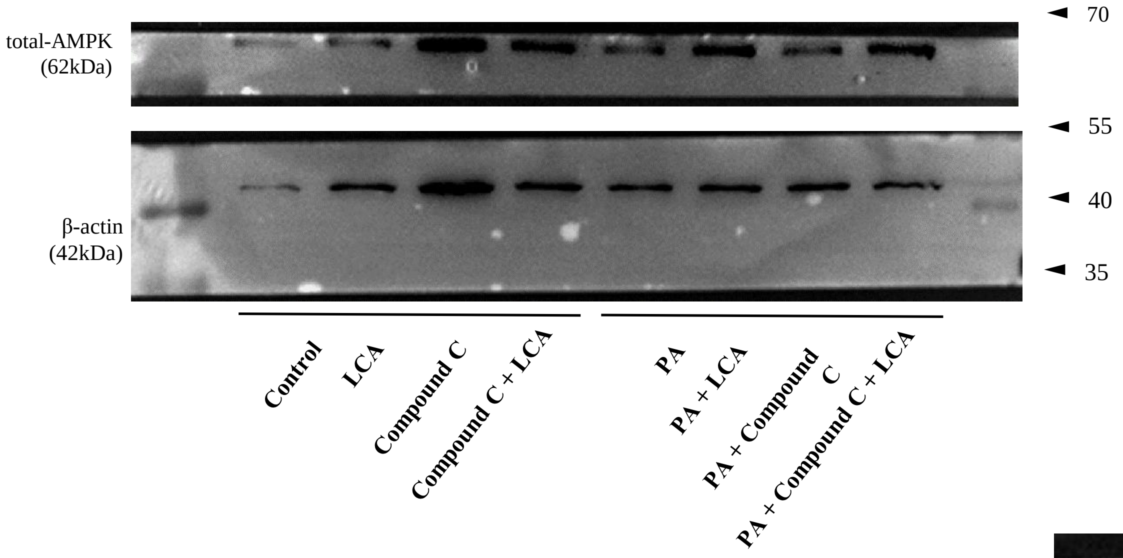
total-AMPK
(62kDa)
β-actin
(42kDa)
70
55
40
35
Control
LCA
Compound C
Compound C + LCA
PA
PA + LCA
PA + Compound C
PA + Compound C + LCA

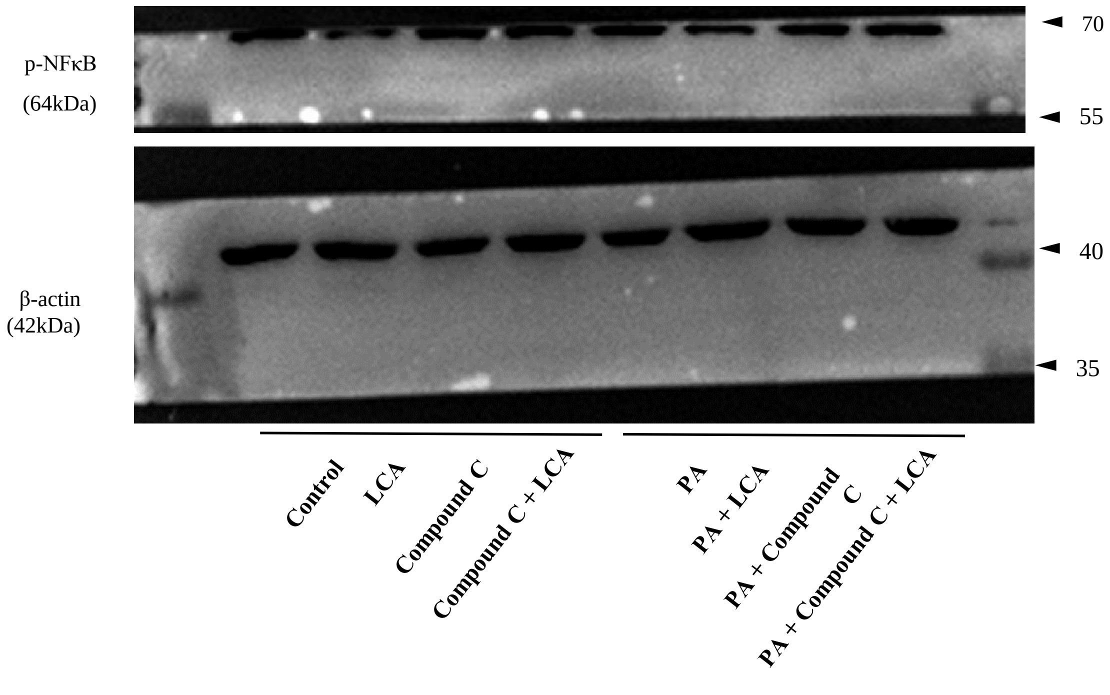
p-NFκB
(64kDa)
β-actin
(42kDa)
70
55
40
35
Control
LCA
Compound C
Compound C + LCA
PA
PA + LCA
PA + Compound C
PA + Compound C + LCA

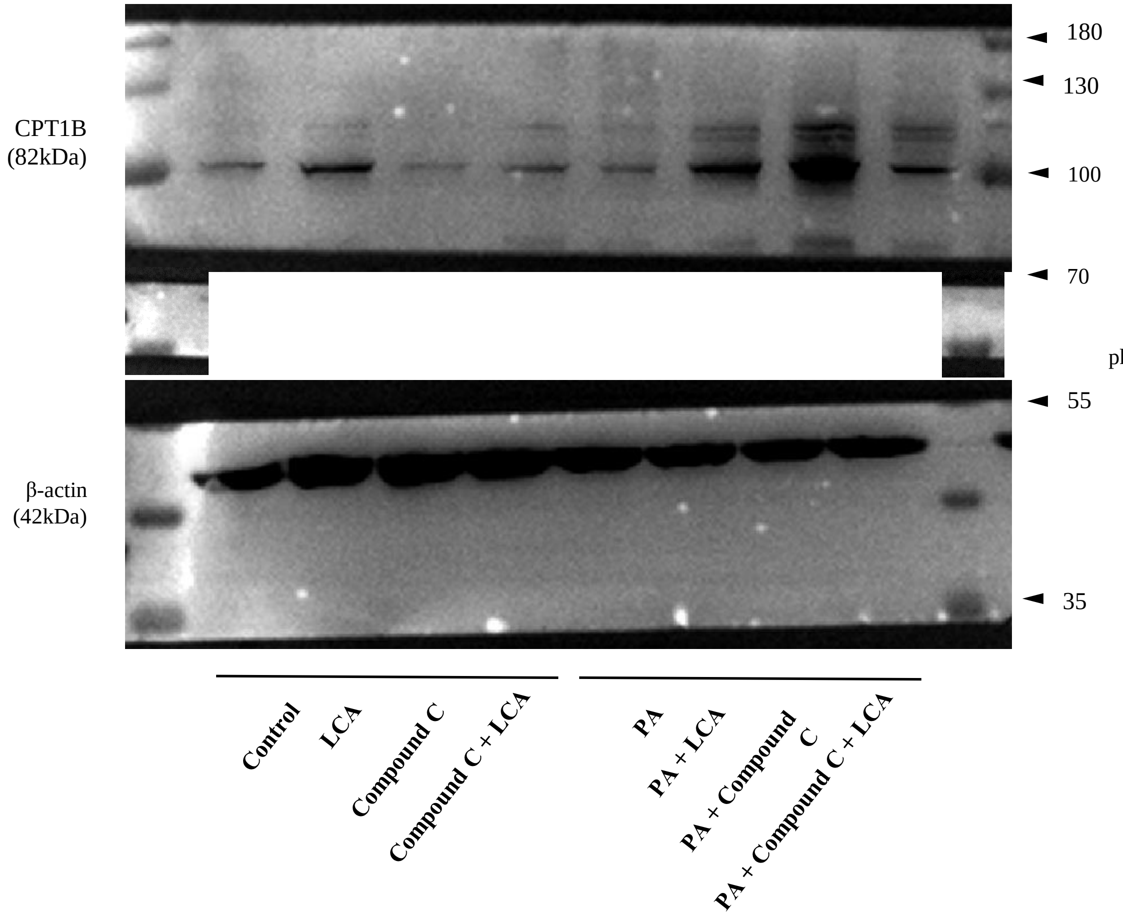
CPT1B
(82kDa)
β-actin
(42kDa)
180
130
100
70
55
35
Control
LCA
Compound C
Compound C + LCA
PA
PA + LCA
PA + Compound C
PA + Compound C + LCA

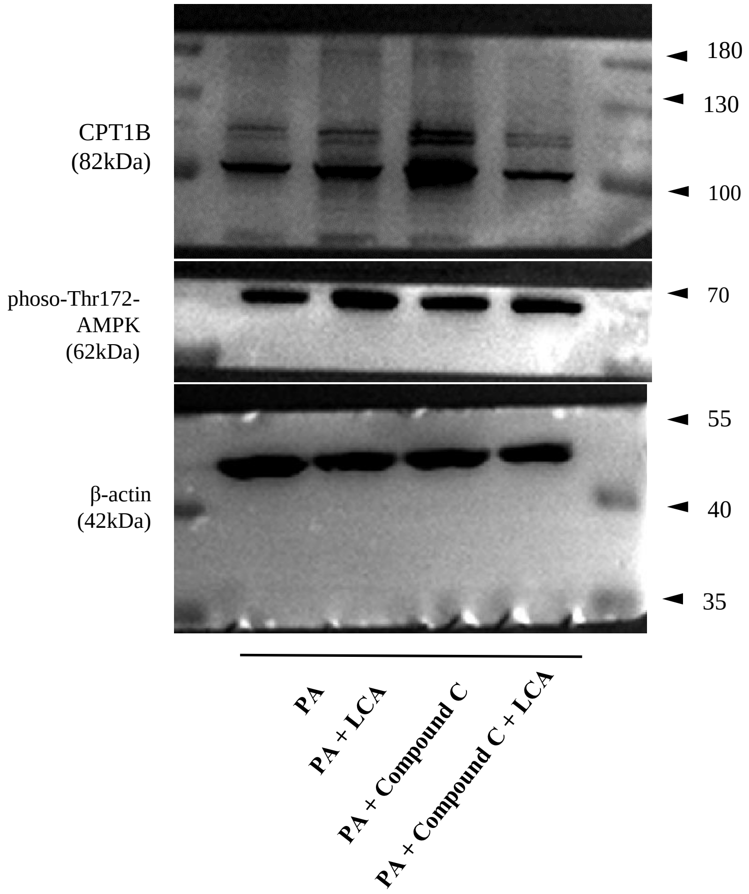
CPT1B
(82kDa)
phoso-Thr172-AMPK
(62kDa)
β-actin
(42kDa)
180
130
100
70
55
40
35
PA
PA + LCA
PA + Compound C
PA + Compound C + LCA

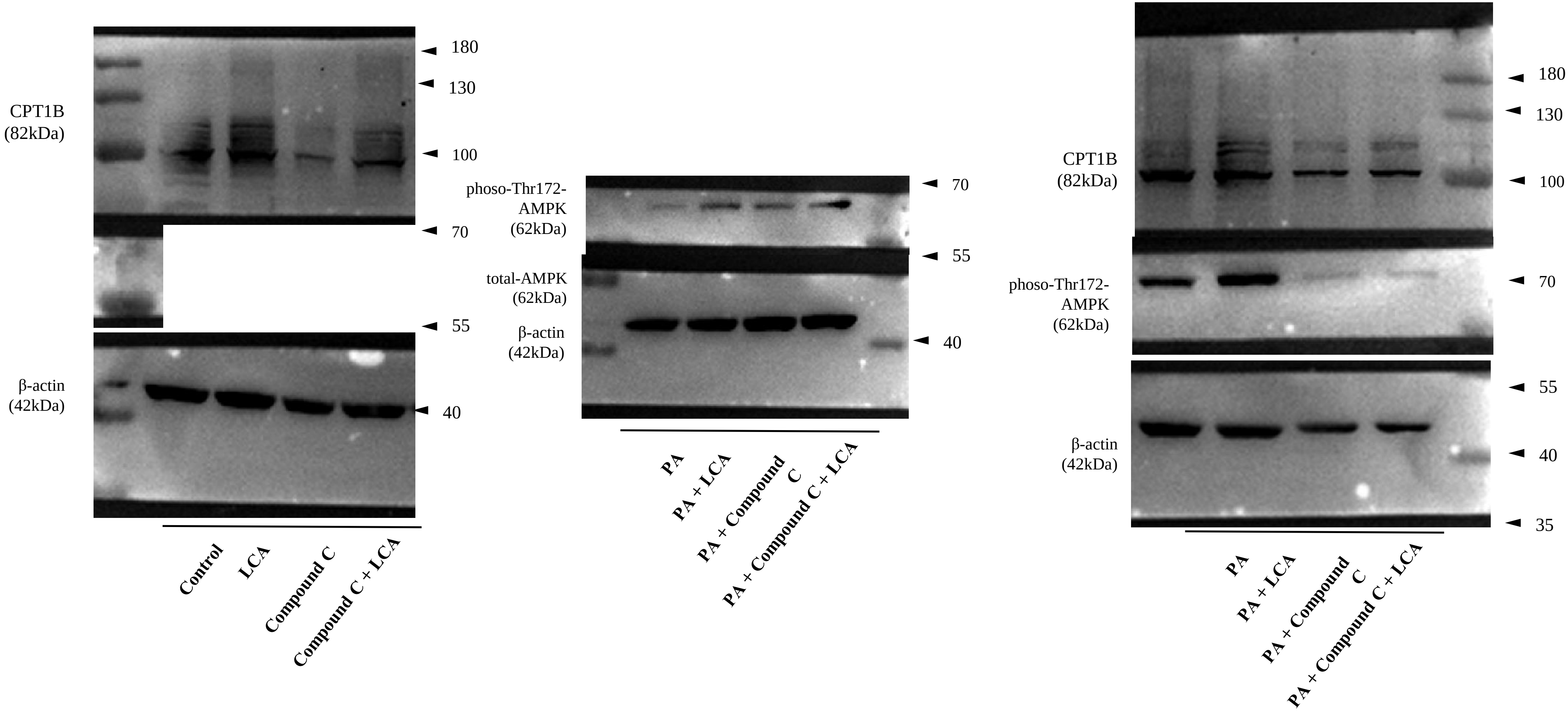
CPT1B
(82kDa)
β-actin
(42kDa)
180
130
100
70
55
40
Control
LCA
Compound C
Compound C + LCA
phoso-Thr172-
AMPK
(62kDa)
total-AMPK
(62kDa)
β-actin
(42kDa)
70
55
40
PA
PA + LCA
PA + Compound
C
PA + Compound C + LCA
CPT1B
(82kDa)
phoso-Thr172-
AMPK
(62kDa)
β-actin
(42kDa)
180
130
100
70
55
40
35
PA
PA + LCA
PA + Compound
C
PA + Compound C + LCA

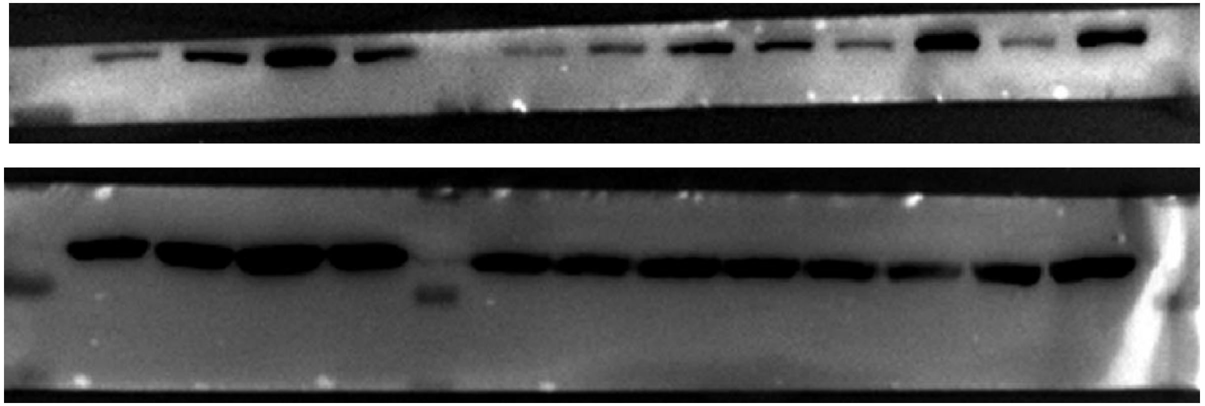
phoso-Thr172-
AMPK
(62kDa)
β-actin
(42kDa)
70
55
40
Control
LCA
Compound C
Compound C + LCA
Control
LCA
Compound C
Compound C + LCA
PA
PA + LCA
PA + Compound
C
PA + Compound C + LCA

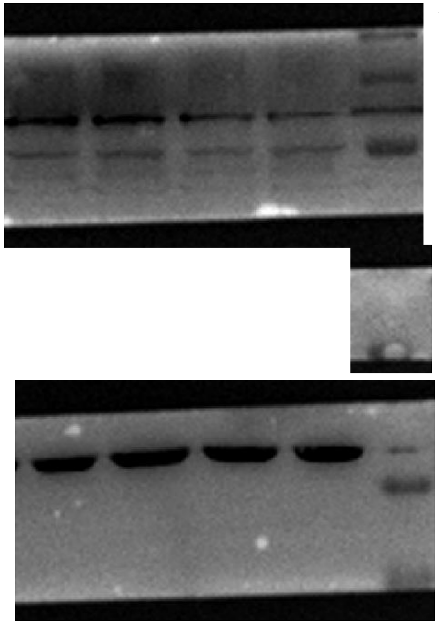
CPT1B
(82kDa)
β-actin
(42kDa)
180
130
100
70
55
40
35
Control
LCA
Compound C
Compound C + LCA

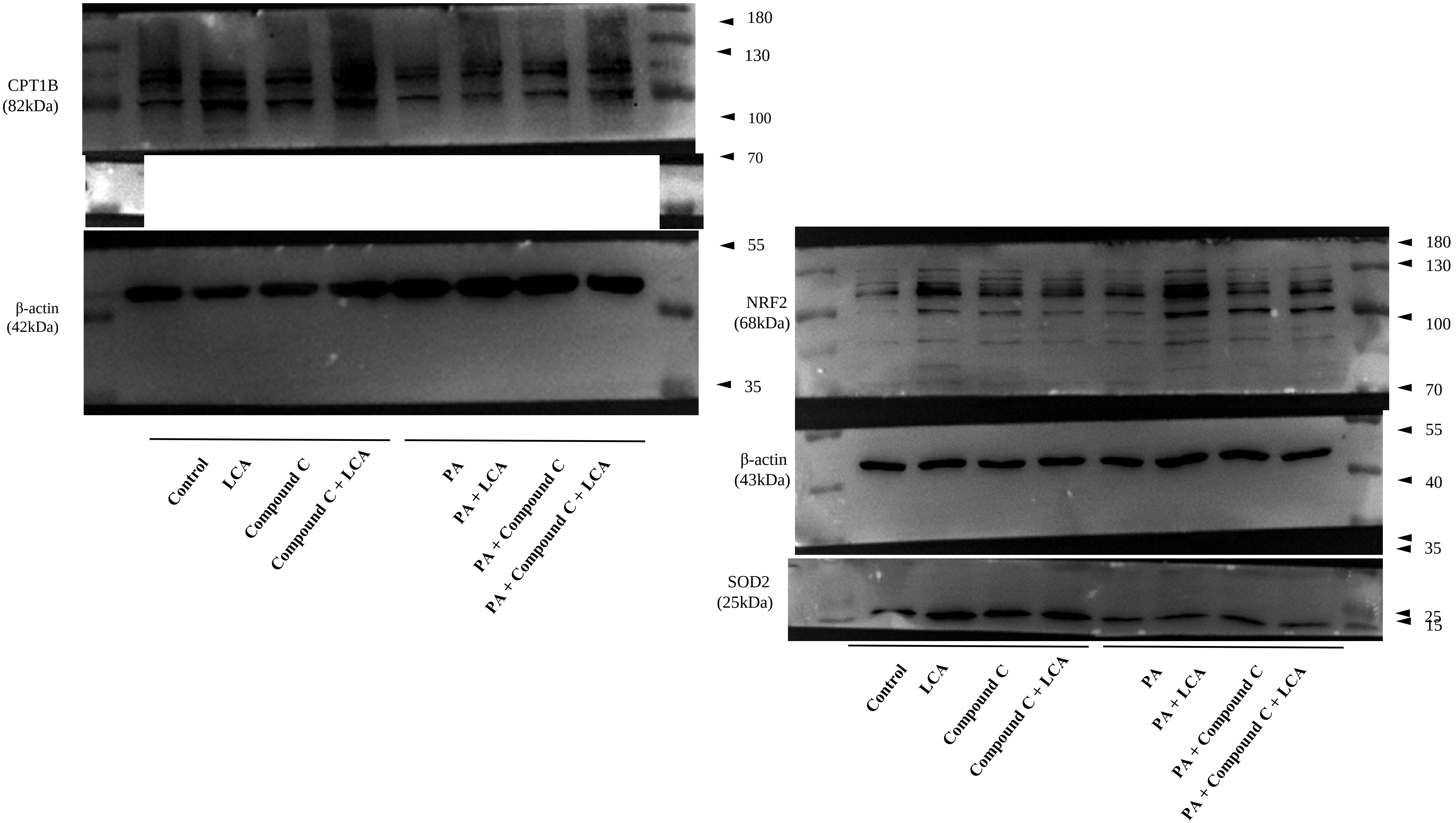
CPT1B
(82kDa)
180
130
100
70
55
35
β-actin
(42kDa)
Control
LCA
Compound C
Compound C + LCA
PA
PA + LCA
PA + Compound C
PA + Compound C + LCA
NRF2
(68kDa)
180
130
100
70
55
40
35
β-actin
(43kDa)
SOD2
(25kDa)
25
15
Control
LCA
Compound C
Compound C + LCA
PA
PA + LCA
PA + Compound C
PA + Compound C + LCA

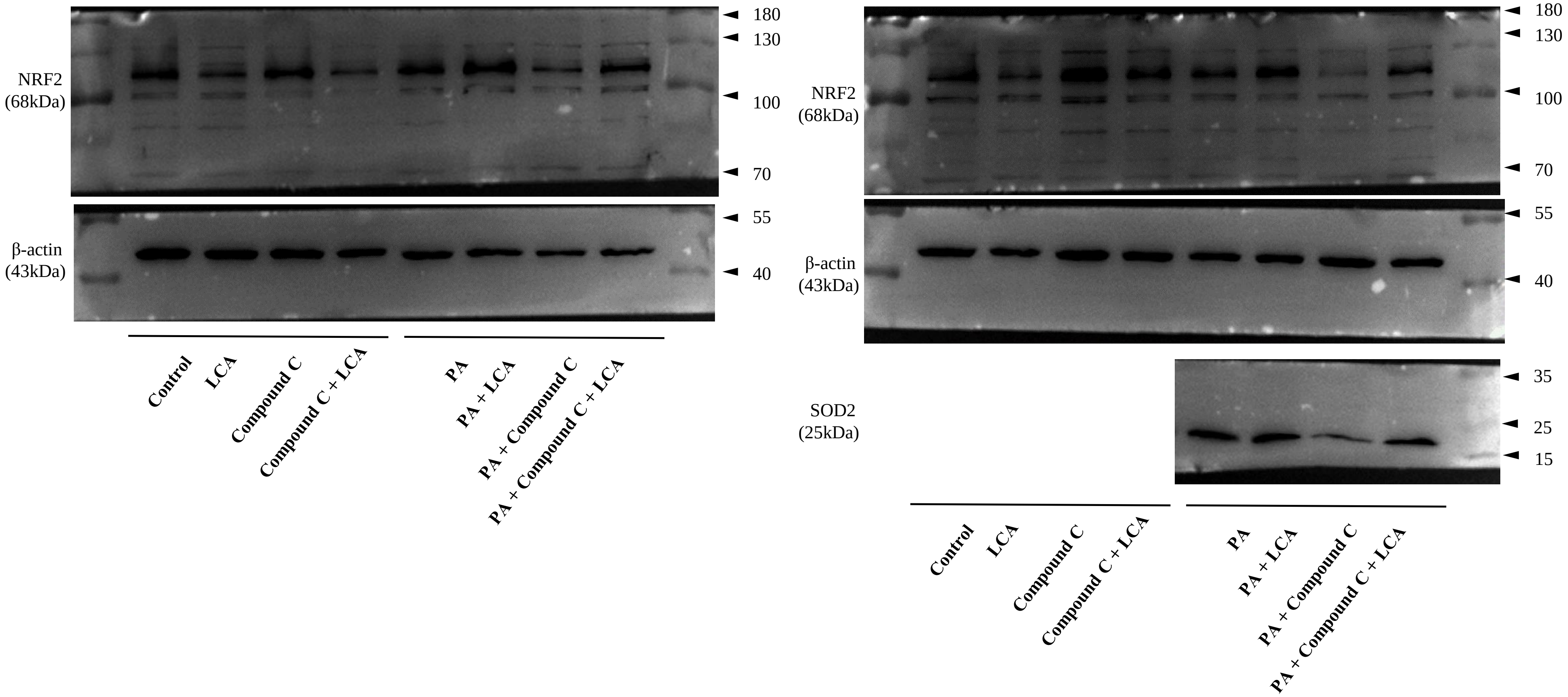

NRF2
(68kDa)
β-actin
(43kDa)
180
130
100
70
55
40
Control
LCA
Compound C
Compound C + LCA
PA
PA + LCA
PA + Compound C
PA + Compound C + LCA
NRF2
(68kDa)
β-actin
(43kDa)
SOD2
(25kDa)
180
130
100
70
55
40
35
25
15
Control
LCA
Compound C
Compound C + LCA
PA
PA + LCA
PA + Compound C
PA + Compound C + LCA

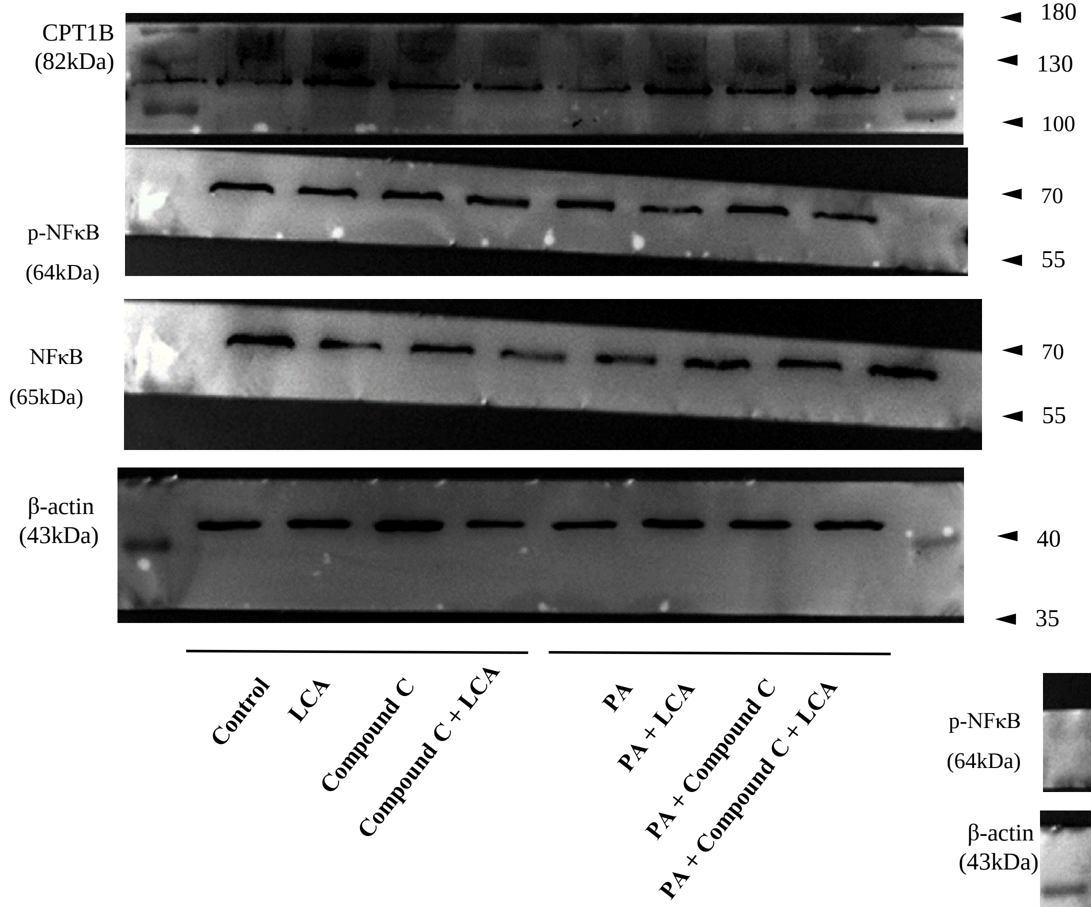
CPT1B
(82kDa)
p-NFκB
(64kDa)
NFκB
(65kDa)
β-actin
(43kDa)
180
130
100
70
55
70
55
40
35
Control
LCA
Compound C
Compound C + LCA
PA
PA + LCA
PA + Compound C
PA + Compound C + LCA

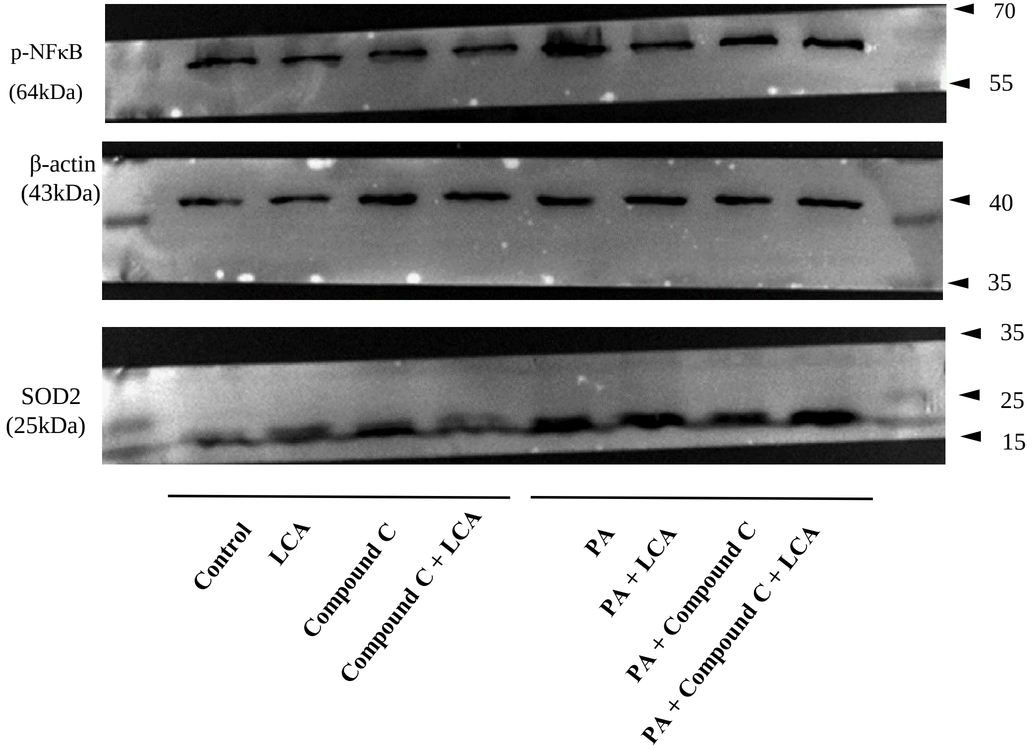
p-NFκB
(64kDa)
β-actin
(43kDa)
SOD2
(25kDa)
70
55
40
35
35
25
15
Control
LCA
Compound C
Compound C + LCA
PA
PA + LCA
PA + Compound C
PA + Compound C + LCA

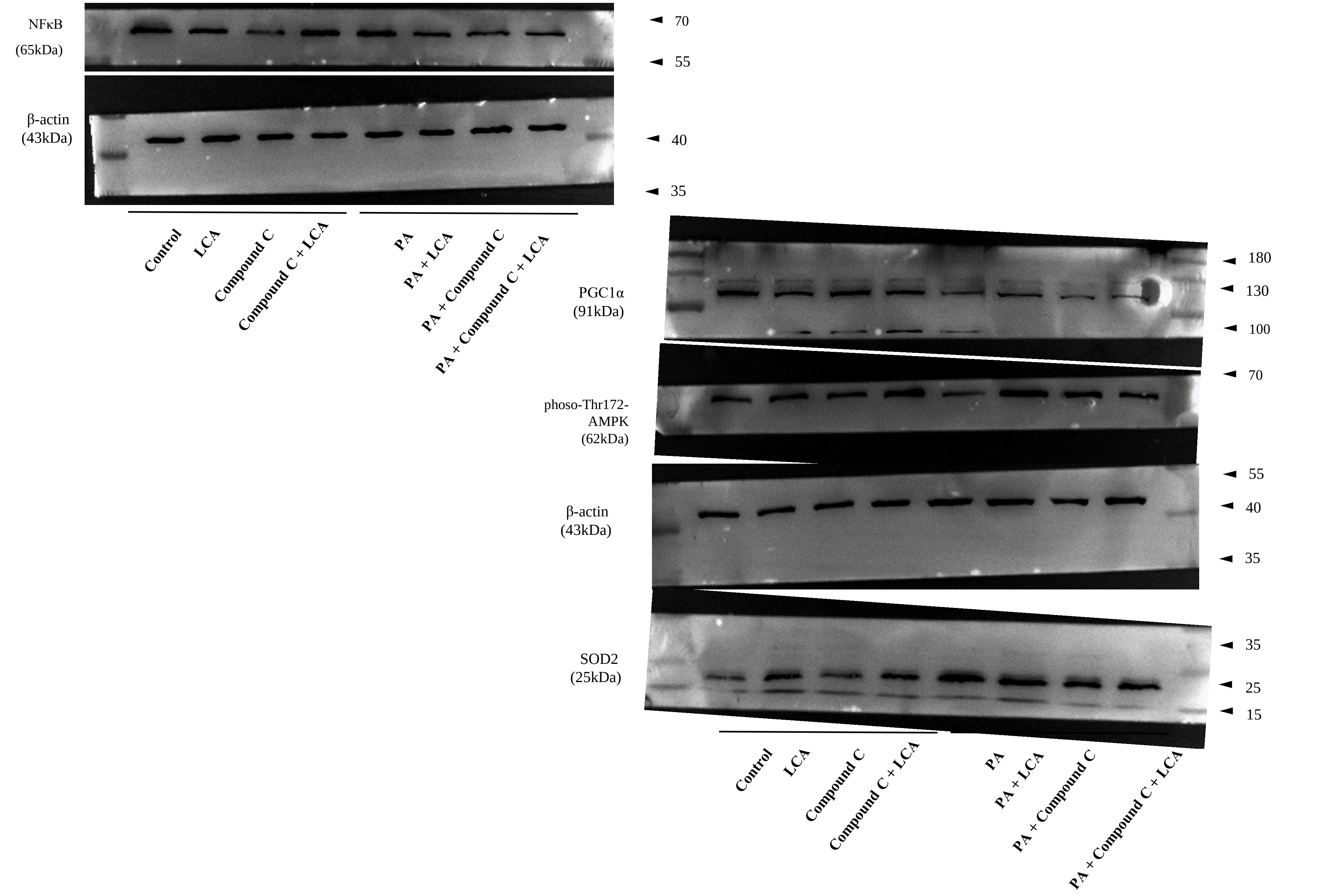

NFκB
(65kDa)
β-actin
(43kDa)
70
55
40
35
Control
LCA
Compound C
Compound C + LCA
PA
PA + LCA
PA + Compound C
PA + Compound C + LCA
PGC1α
(91kDa)
180
130
100
phoso-Thr172-
AMPK
(62kDa)
70
β-actin
(43kDa)
55
40
35
SOD2
(25kDa)
35
25
15
Control
LCA
Compound C
Compound C + LCA
PA
PA + LCA
PA + Compound C
PA + Compound C + LCA

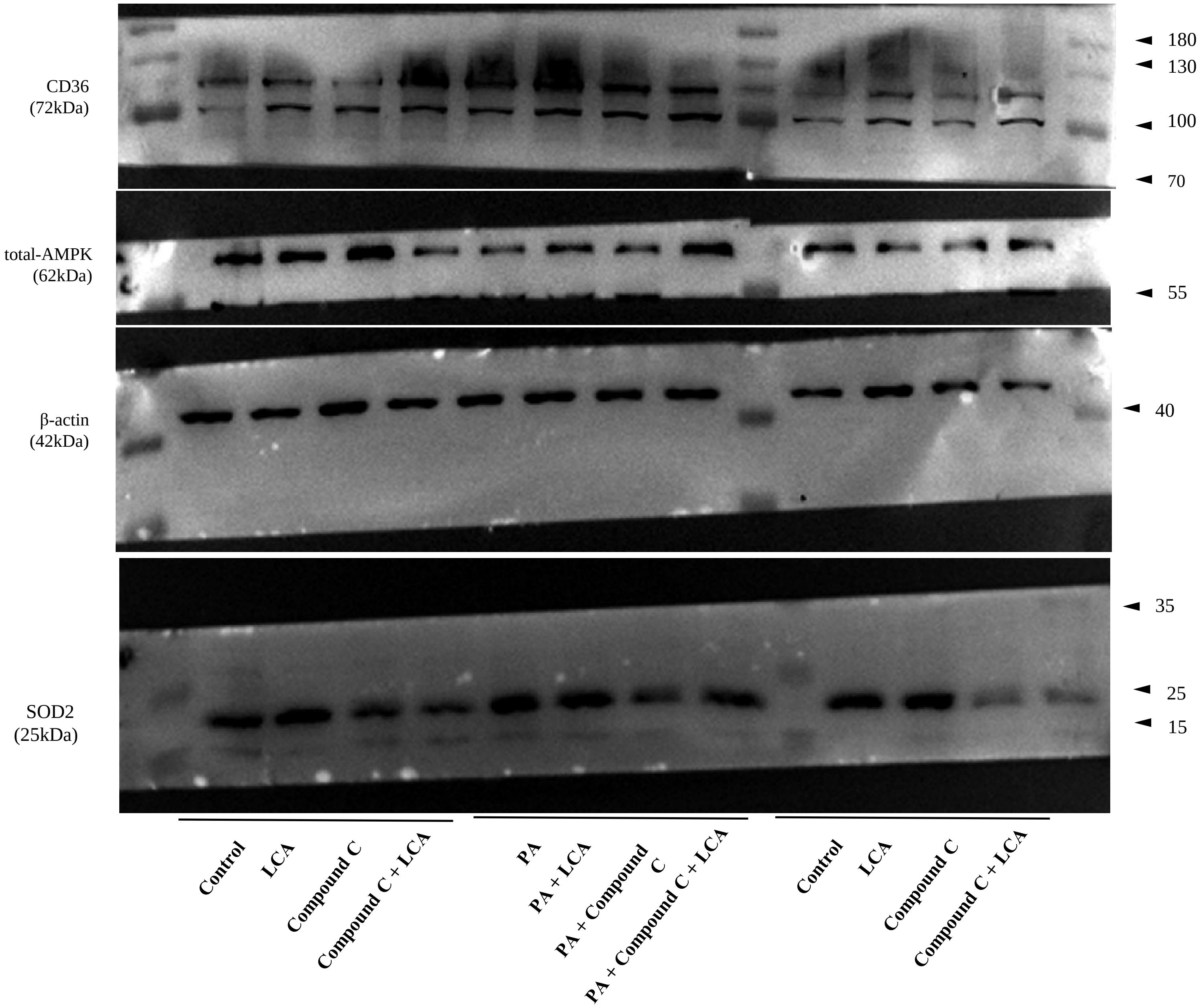
CD36
(72kDa)
total-AMPK
(62kDa)
β-actin
(42kDa)
SOD2
(25kDa)
180
130
100
70
55
40
35
25
15
Control
LCA
Compound C
Compound C + LCA
PA
PA + LCA
PA + Compound C
PA + Compound C + LCA
Control
LCA
Compound C
Compound C + LCA

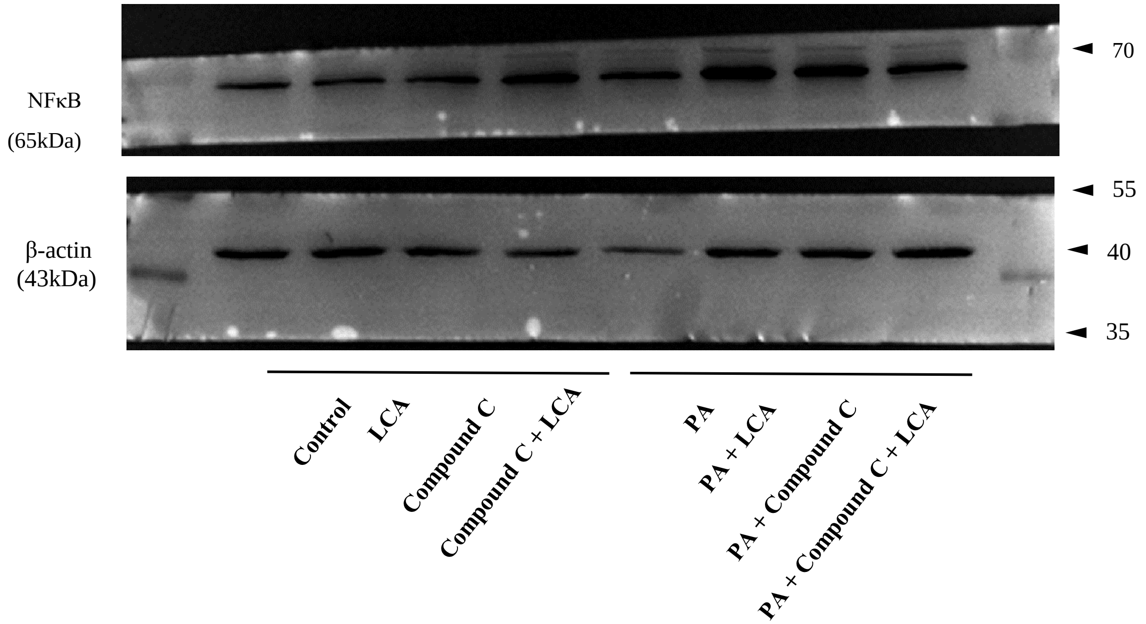
NFκB
(65kDa)
β-actin
(43kDa)
70
55
40
35
Control
LCA
Compound C
Compound C + LCA
PA
PA + LCA
PA + Compound C
PA + Compound C + LCA

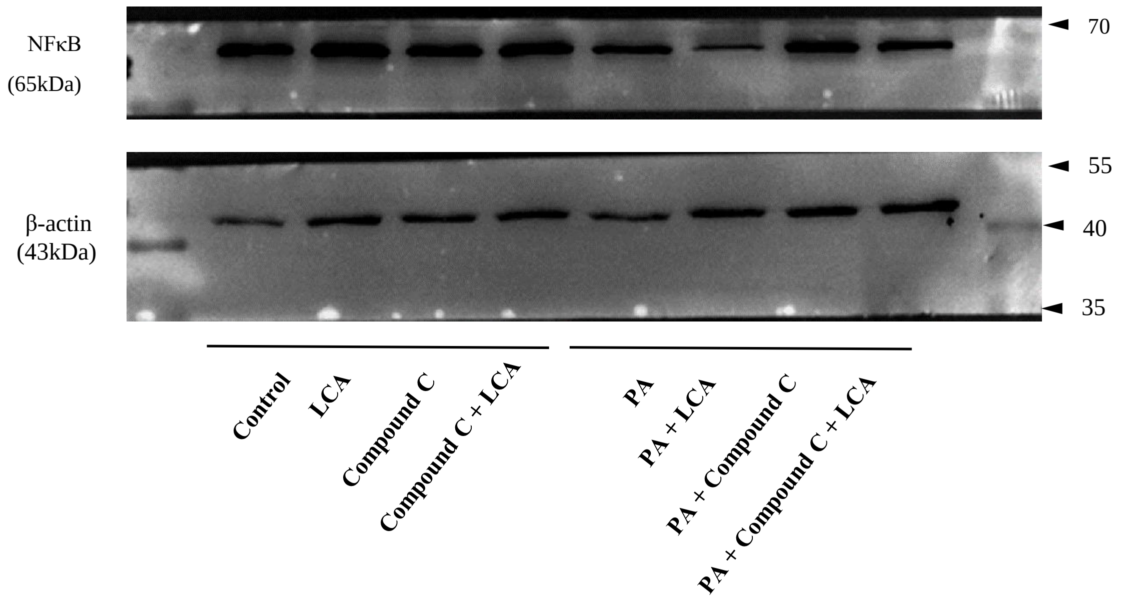
NFκB
(65kDa)
β-actin
(43kDa)
70
55
40
35
Control
LCA
Compound C
Compound C + LCA
PA
PA + LCA
PA + Compound C
PA + Compound C + LCA

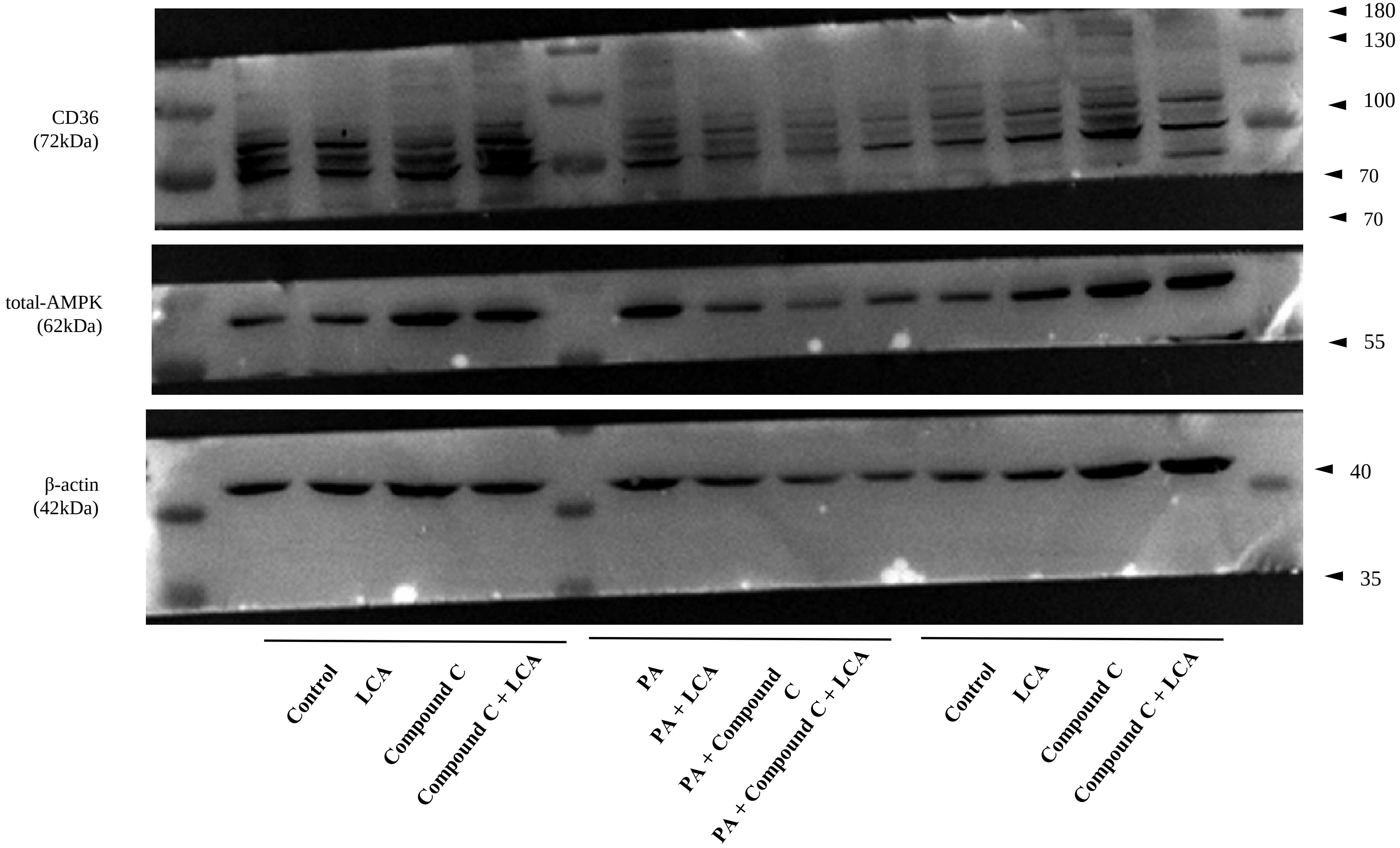
CD36
(72kDa)
total-AMPK
(62kDa)
β-actin
(42kDa)
180
130
100
70
70
55
40
35
Control
LCA
Compound C
Compound C + LCA
PA
PA + LCA
PA + Compound C
PA + Compound C + LCA
Control
LCA
Compound C
Compound C + LCA
